# Physicochemical Dynamics, Microbial Succession, and Volatile Profiles of Light-Flavor Baijiu Fermented Grains Prepared from Different Rice Varieties

**DOI:** 10.3390/foods15213825

**Published:** 2026-10-26

**Authors:** Wenjun Rao, Xin Li, Yaoyao Qi, Shuai Hu, Linghua Ouyang, Shuilan Zhu, Shuhan Yi, Jinying Zhou

**Affiliations:** Institute of Agricultural Products Processing, Jiangxi Academy of Agricultural Sciences, 602 Nanlian Road, Nanchang 330200, China; raowenjun0@163.com (W.R.); lx0514nc@163.com (X.L.); qiyaoyaoer711@163.com (Y.Q.); shuai.hu@jxaas.cn (S.H.); oylh10@126.com (L.O.); zhushuilan88@126.com (S.Z.); yishuhan1@163.com (S.Y.)

**Keywords:** light-flavor baijiu, fermented grains, microbial community succession, rice varieties

## Abstract

Light-flavor baijiu is a traditional Chinese liquor, and its flavor formation depends on the dynamic succession and metabolic regulation of the complex microbial community within the fermented grains. This study aims to elucidate the mechanisms by which different rice varieties influence the fermentation process of light-flavor baijiu fermented grains. Three representative rice varieties (glutinous rice, japonica rice and indica rice) were selected as raw materials, and the physicochemical properties, microbial community succession, and changes in flavor metabolites during 35-day fermentation were systematically monitored. The results showed that distinct dynamic patterns of physicochemical indicators including temperature, moisture, acidity, sugar content and alcohol were observed in fermented grains from different rice varieties, and differences in starch composition may be one important contributing factor for these variations. High-throughput sequencing analysis revealed that the bacterial community was dominated by *Firmicutes* and *Proteobacteria*, while *Ascomycota* accounted for most of the fungal community. The successional trajectories at the genus level exhibited clear raw material dependence. pH and starch content were key environmental factors associated with early-stage microbial community assembly, whereas temperature, moisture, acidity, and alcohol content were strongly related to the directional succession of the community in later stages. Flavor metabolite analysis indicated that key volatile compounds such as esters, aldehydes, and ketones accumulated in a stage-specific manner, and their changes were significantly correlated with specific functional microorganisms. This study elucidates that rice varieties influence the microecological environment, community succession patterns, and flavor-metabolite trajectories of the fermented grains, which represent flavor potential for subsequent distillation. It provides a theoretical basis for raw material selection and fermentation optimization in light-flavor baijiu production.

## 1. Introduction

Baijiu is a traditional Chinese distilled spirit that plays a significant role in both culinary culture and social occasions. Among its aroma types, the light-flavor baijiu is characterized by a clean and a relatively delicate fruity and floral aroma profile [1,2]. Light-flavor baijiu is manufactured via solid-state fermentation followed by distillation. Daqu serves as a complex natural microbial starter supplying saccharifying and fermentative microbes, and most aroma precursors are generated within fermented grains (jiupei), which are partially transferred into final liquor during distillation. The formation of its unique flavor profile occurs primarily in the fermented grains (jiupei), where microorganisms introduced with the Daqu (starter) and processing environment interact with the substrate under continuously changing physicochemical conditions. Previous investigations have demonstrated that key bacterial and fungal taxa in fermented grains are closely associated with starch degradation, ethanol production, and the accumulation of alcohols, acids, esters, and other compounds that contribute to the aroma and quality of the distilled product [3,4]. Accordingly, unraveling the dynamic relationship among substrate properties, fermentation environment, microbial community structure, and flavor metabolites is important for understanding and improving the quality of light-flavor baijiu.

The brewing raw materials represent a key upstream factor of this process because it provides not only fermentable carbon and nutrients but also the physicochemical matrix within which microbial growth and metabolism occur [5]. Rice provides an alternative brewing substrate in light-flavor baijiu fermentation systems and is of interest because of its high starch content, broad varietal diversity, and abundant regional availability. Recent studies have demonstrated that rice varietal differences can substantially modify fermentation behavior. For example, multi-omics comparison of different glutinous rice varieties revealed pronounced differences in microbial regulation and metabolic-flavor trajectories during baijiu fermentation [6], while Rao et al. further showed that rice raw-material characteristics were associated with distinct key aroma compound profiles in rice-base liquor [7]. However, compared with sorghum, the influence of rice varietal characteristics on the fermentation ecology of light-flavor baijiu is still poorly resolved. Most existing studies have largely focused on a specific raw material or final liquor characteristics, while systematic time-resolved evidence linking rice substrate variation to the coupled development of the physicochemical environment, microbial communities, and volatile metabolites in light-flavor fermented grains remains limited [8].

Accordingly, this study compared three representative rice varieties under a common light-flavor baijiu fermentation process. We investigated the temporal relationships among physicochemical changes, bacterial and fungal succession, and volatile-metabolite development throughout fermentation. Through integrated analysis, this study aims to clarify how differences among rice substrates are associated with the dynamic fermentation ecosystem and flavor formation. This work will provide a framework for understanding the raw material-associated variation within the fermentation ecosystem and may inform rational rice selection and fermentation strategy for rice-based light-flavor baijiu.

## 2. Materials and Methods

### 2.1. Materials and Fermentation Design

The three rice varieties were provided by the Rice Research Institute of the Jiangxi Academy of Agricultural Sciences: Ganningjing No. 1 (glutinous rice, GR), Jinggangshan Rice (indica rice, IR), and Ganwanjing No. 688 (japonica rice, JR). The chemical composition of the three rice varieties was summarized in Table S1. Light-flavor low-temperature Daqu was purchased from Fenyuan Daqu Manufacturing Co., Ltd. (Fenyang, China) and the same batch of Daqu was used for all fermentation treatments.

Light-flavor baijiu brewing was carried out using a typical solid-state fermentation. For each rice variety, 125 kg of rice was soaked in water for 12 h at 25 °C, followed by steaming for 30 min. After cooling at 25 to 28 °C, the steamed rice was uniformly mixed with 10% Daqu (*w*/*w*) and then transferred into 1000 L pottery jars at 125 kg per jar. Each rice treatment comprised 3 independent fermentation jars. The fermentation was conducted for 35 d under 25 °C. Fermented-grain samples were collected on days 0, 2, 7, 14, 21, 28, and 35. Sampling from fermentation jars: The vertical direction was divided into upper, middle, and lower layers, each with a thickness of 40 cm. Six sampling points were set in each layer, and 30 g of sample was collected from each point. After the samples from all points were collected, they were thoroughly mixed to obtain a composite sample representing that jar, which was used for subsequent detection and analysis. All samples collected at the seven time points were used for physicochemical analyses. Based on the results of these analyses, samples from days 0, 2, 14, and 35 were additionally selected for electronic nose analysis, volatile compound profiling, and microbial community analysis. All samples were stored at −80 °C until analysis.

### 2.2. Determination of Physicochemical Properties in Fermented Grains

Fermentation temperature, moisture, pH, acidity, starch content, reducing-sugar content, and alcohol content were determined at the designated sampling points. Temperature was monitored in real time using a digital temperature sensor (Lcd-105, Hengshui Zhengxu Electronic Technology Co., Ltd., Hengshui, China) positioned at 80 cm depth within each fermentation jar. Moisture content was determined using an HE53 moisture analyzer (Mettler-Toledo Instruments Co., Ltd., Shanghai, China). For pH determination, 1 g of fermented grains was homogenized with 3 mL of distilled water, and the pH of resulting suspension was measured using a calibrated pH meter (pH-30, Shanghai Yidian Scientific Instrument Co., Ltd., Shanghai, China). Titratable acidity was analyzed by titration with 0.01 M NaOH solution and expressed as mmol NaOH/10 g fermented grains. Starch content was determined according to the acid hydrolysis method described by Tang et al. Briefly, starch content was calculated by calculating the difference between the total reducing sugar and the original reducing sugar after hydrolysis with HCl (20%, *v*/*v*) for 30 min, and results were expressed as mg/g of fermented grains [9]. Reducing sugar content was measured using the 3,5-dinitrosalicylic acid (DNS) method and expressed as mg/g fermented grains [10]. Alcohol content was analyzed by distillation followed by alcoholmeter measurement [11]. Briefly, 100 g of fermented grains was mixed with 100 mL of distilled water and distilled, and 50 mL of distillate was collected. The alcohol content was measured using an alcoholmeter (lx054, Changzhou Ruiming Instrument Factory, Changzhou, China) at 25 °C.

### 2.3. Determination of Volatile Compounds in Fermented Grains

#### 2.3.1. HS-SPME Extraction of Volatile Compounds

Volatile compounds in the fermented grains were extracted by headspace solid-phase microextraction (HS-SPME). Briefly, 2 g of homogenized fermented grains was transferred into a 20 mL headspace vial containing 5 mL of saturated NaCl solution, and 10 μL of 2-octanol solution (158 mg/L, aladdin, Shanghai Aladdin Biochemical Technology Co., Ltd., Shanghai, China) was added as the internal standard. The vial was immediately sealed and equilibrated at 60 °C for 20 min, followed by extraction at 60 °C for 10 min using a 50/30 μm DVB/CAR/PDMS SPME fiber (Sigma-Aldrich Co. LLC, St. Louis, MO, USA). Before analysis, the extraction device was conditioned at 250 °C for 30 min. After extraction, the fiber was immediately introduced into the GC injection port and thermally desorbed at 250 °C for 3 min.

#### 2.3.2. GC-MS Analysis

GC-MS analysis was performed using a GCMS-TQ8040NX system equipped with an AOC-6000 Plus autosampler (Shimadzu, Shimadzu Enterprise Management, China Co., Ltd., Shanghai China). Volatile compounds were separated on an SH-Wax column (30 m × 0.25 mm × 0.25 μm, Shimadzu, Shanghai China). Helium (99.999%) was used as the carrier gas at a constant flow rate of 1.0 mL/min in splitless injection mode. The oven temperature program was as follows: initial temperature of 50 °C held for 5 min, then increased to 250 °C at a rate of 10 °C/min and held for 10 min. The mass spectrometer was operated in electron ionization (EI) mode at 70 eV, with an ion source temperature of 200 °C and a quadrupole temperature of 150 °C. The mass scan range was set from 30 to 500 *m*/*z*. Volatile compounds were putatively identified by matching their mass spectra against the NIST 20 library, and only matches with similarity scores ≥ 800 were retained. Volatile compounds were semi-quantified using 2-octanol as the internal standard based on analyte to internal standard peak-area ratios.

### 2.4. Electronic Nose Analysis

Electronic nose profiling was performed using a PEN3 electronic nose (Airsense, Schwerin, Germany) equipped with ten metal-oxide semiconductor sensors (W1C, W5S, W3C, W6S, W5C, W1S, W1W, W2S, W2W, and W3S), following Tu et al. with slight modifications [12]. For each measurement, 3 g of fermented grains was placed in a 100 mL vial, immediately sealed, and equilibrated at 60 °C for 30 min. The sensor chamber was flushed with clean air for 80 s and zero-adjusted for 5 s. After a 5 s sample-preparation period, headspace gas was introduced into the sensor chamber at a flow rate of 400 mL/min, and sensor responses were recorded for 80 s and used for subsequent analysis.

### 2.5. Analysis of Microbial Communities in Fermented Grains

Total microbial DNA was extracted using the E.Z.N.A.^®^ soil DNA Kit (Omega Bio-tek, Norcross, GA, USA) following the manufacturer’s instructions with slight modifications based on Han et al. [13]. The V3-V4 region of the bacterial 16S rRNA gene was amplified with the primers 338F (ACTCCTACGGGAGGCAGCAG) and 806R (GGACTACHVGGGTWTCTAAT). The fungal ITS1 region was amplified using the primers F-CTTGGTCATTTAGAGGAAGTAA and R-GCTGCGTTCTTCATCGATGC. High-throughput sequencing was performed on an Illumina Nextseq2000 platform by Shanghai Majorbio Bio-pharm Technology Co., Ltd. (Shanghai, China). Raw sequencing reads were quality-controlled using fastp (version 0.19.6) assembled using FLASH (version 1.2.11), and processed using UPARSE (version 11.0.667). After chimeric sequences, sequences were clustered into operational taxonomic units (OTUs) at 97% similarity. Representative bacterial and fungal sequences were taxonomically classified against the SILVA Release 138 and UNITE version 8.0, respectively, using a 70% confidence threshold. Because SILVA Release 138 predates the 2020 taxonomic revision of Lactobacillus, the Lactobacillus label in our OUT-based classification follows the traditional pre-2020 nomenclature and may include taxa subsequently reclassified into genera such as *Lacticaseibacillus*, *Lentilactobacillus*, and *Latilactobacillus*. Good’s coverage, Chao1 richness, Shannon diversity, and taxonomic relative abundances were calculated using the Majorbio Cloud Platform (https://cloud.majorbio.com).

### 2.6. Statistical Analysis

Data visualization was performed using Origin 2021 and GraphPad Prism 9.5.0. Results are presented as mean ± standard deviation (SD) from 3 independent biological replicates, unless otherwise stated. Differences among samples were evaluated using one-way Analysis of Variance (ANOVA), followed by Tukey’s post-hoc test, with *p* < 0.05 considered statistically significant. Principal component analysis (PCA) of volatile compound profiles was performed using SIMCA 14.1 software (Sartorius Stedim Data Analytics AB, Umeå, Sweden). PLS-DA models with 7-fold cross-validation were subsequently constructed to identify variables contributing to group discrimination. Variables with VIP > 1 were considered important contributors to model discrimination. For PCA, PLS-DA and correlation analysis, compounds below the detection limit (marked as ND in Supplementary Tables) were assigned a value of zero for calculation. Redundancy analysis (RDA) was used to examine associations between physicochemical variables and microbial-community composition, and 200 permutations were used to evaluate model over-fitting. Associations among microbial taxa and between microbial genera and volatile compounds were evaluated using Pearson correlation method controlled using the Benjamini Hochberg false discovery rate (FDR) procedure. Only pairs with |r| ≥ 0.6 and FDR-adjusted *p* < 0.05 were retained for network construction. Co-occurrence networks were constructed and visualized using Gephi software 0.9.2. Keystone nodes were defined based on high betweenness centrality and high degree values from network topological parameters, where taxa exhibiting relatively high degree and high betweenness centrality were regarded as potential keystone nodes. 

## 3. Results

### 3.1. Physicochemical Dynamics During Fermentation

**Figure 1:**
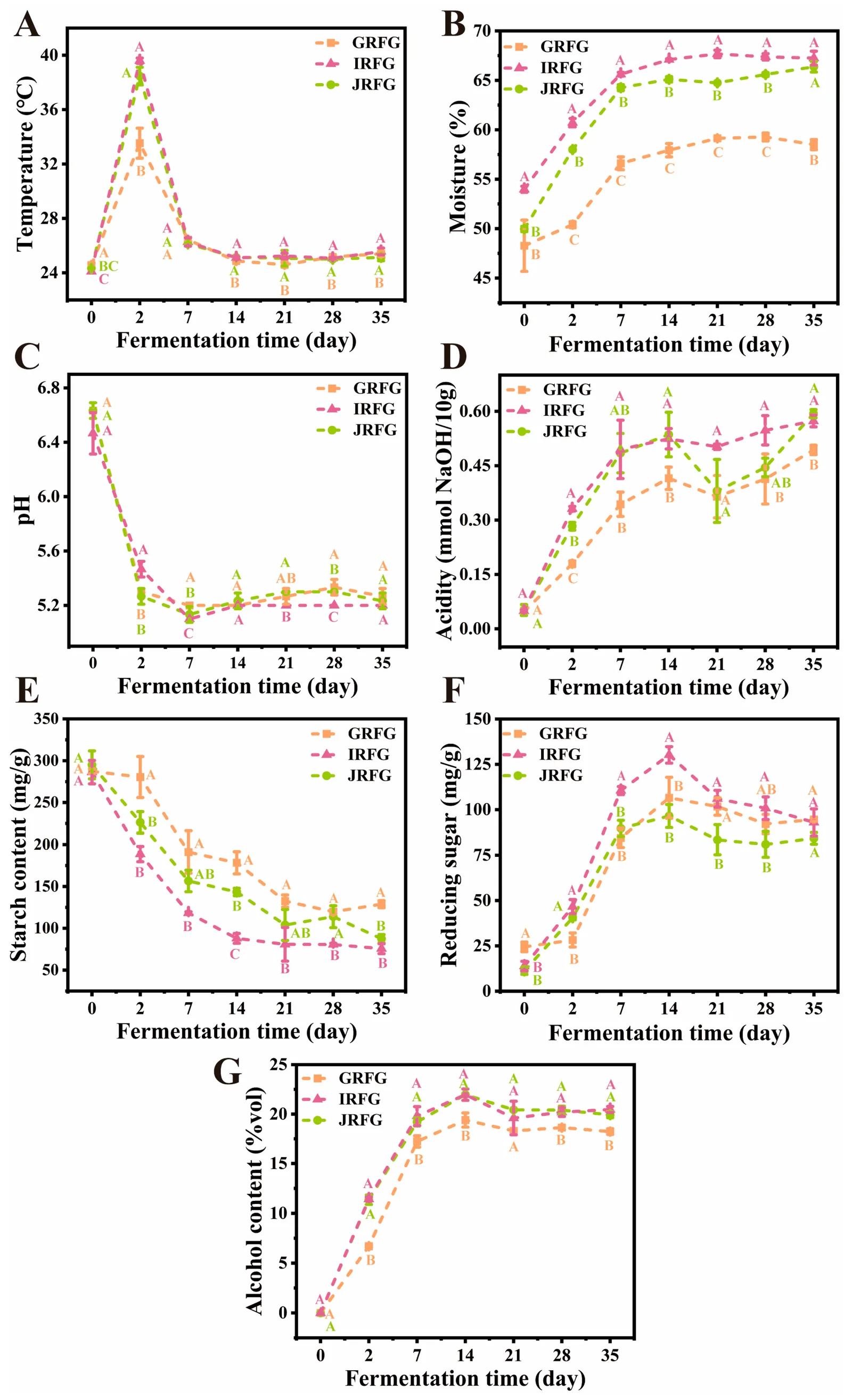
Physicochemical dynamics of fermented grains prepared from glutinous rice (GR), indica rice (IR), and japonica rice (JR) during 35 days of light-flavor baijiu fermentation. (**A**) Fermentation temperature; (**B**) moisture content; (**C**) pH; (**D**) titratable acidity; (**E**) starch content; (**F**) reducing-sugar content; and (**G**) alcohol content. Different uppercase letters indicate significant differences among rice groups within the same fermentation stage (*p* < 0.05, one-way ANOVA followed by Tukey post-hoc test).

The dynamic changes in physicochemical factors within the fermented grains were monitored over the 35-day fermentation. As shown in Figure 1, the three rice groups exhibited broadly similar physicochemical trajectories, although the magnitude and timing of several changes differed among GR, IR, and JR. Fermentation temperature increased rapidly and peaked on day 2, then dropped sharply before gradually stabilizing after day 14. GR showed a significantly lower peak temperature than IR and JR (Figure 1A). The moisture content gradually increased mainly during the first 14 days and subsequently changed more slowly, with GR maintaining lower moisture levels than the other two groups (Figure 1B). The coordinated early increases in temperature and moisture indicated intense early microbial activity in the initial stage of solid-state fermentation. The acidification profiles also changed markedly over time. The pH dropped sharply within the first 7 days and then remained comparatively stable (Figure 1C). Titratable acidity showed a non-monotonic pattern, increasing during early fermentation (14 days), declining transiently at 21 days, and increasing again toward the later stage (Figure 1D). Changes in carbohydrate utilization were more directly indicative of fermentation progression. The starch content declined continuously in all three rice groups (Figure 1E), whereas reducing sugar accumulated during the early stages (14 days) and subsequently decreased (Figure 1F). Alcohol content increased rapidly during the early stage (14 days) and subsequently approached a plateau (Figure 1G).

Taken together, these physicochemical properties suggest a common dynamic behavior of early intensive transformation followed by a comparatively stable later phase, while rice variety modified the rate and magnitude of substrate conversion and environmental change.

### 3.2. Electronic Nose Analysis During Fermentation

**Figure 2:**
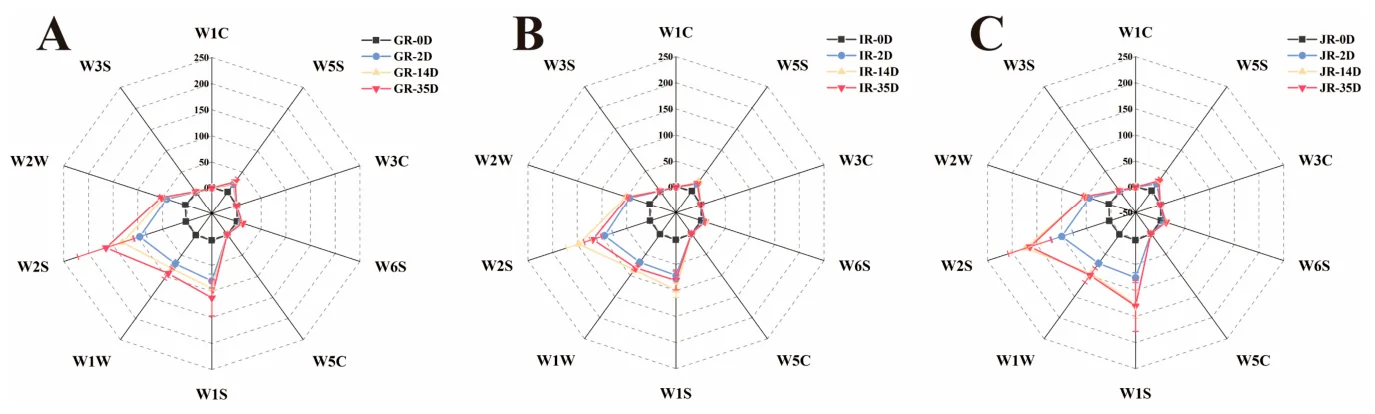
Electronic-nose fingerprints of fermented grains during light-flavor baijiu fermentation. Sensor-response profiles are shown for (**A**) glutinous rice (GR), (**B**) indica rice (IR), and (**C**) japonica rice (JR) at 0 (0D), 2 (2D), 14 (14D), and 35 (35D) days of fermentation.

As shown in Figure 2, electronic nose profiles changed progressively during fermentation in all three rice groups. Although the overall shapes of radar plots were broadly similar across different rice varieties, the intensity of sensor responses varied with rice variety. Among them, sensors W2S, W1S, W1W, and W2W showed comparatively strong responses. As fermentation progressed, the response values of all sensors gradually increased.

### 3.3. Changes in Volatile Compounds of Fermented Grains

**Figure 3:**
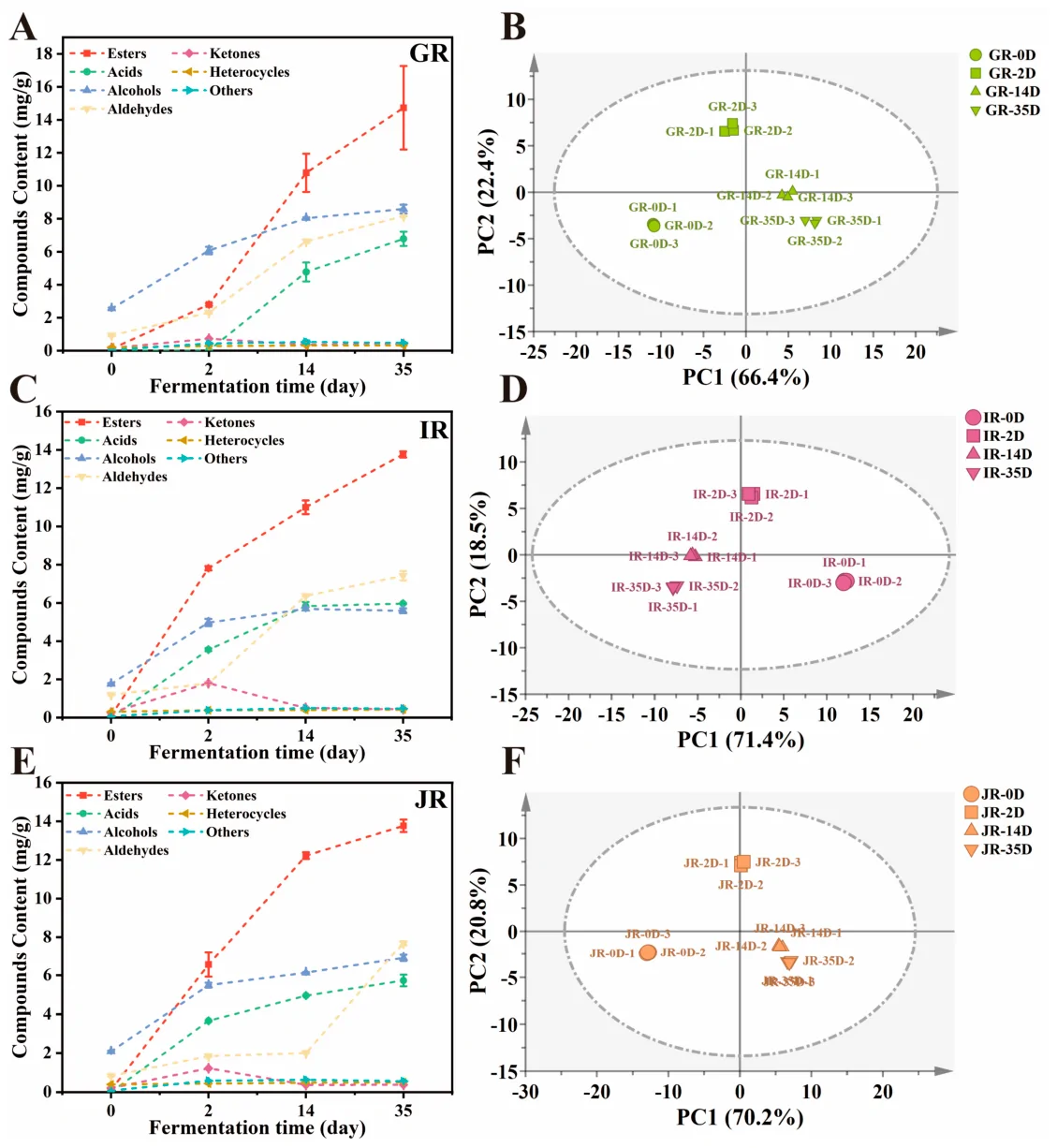
Dynamics of volatile-compound classes and principal component analysis (PCA) of volatile profiles during fermentation. (**A**,**B**) Glutinous-rice fermentation (GR); (**C**,**D**) indica-rice fermentation (IR); and (**E**,**F**) japonica-rice fermentation (JR). Panels (**A**,**C**,**E**) show the semi-quantitative levels of volatile compounds grouped by chemical class, whereas panels (**B**,**D**,**F**) show PCA score plots based on the complete volatile profiles within each rice group.

GC-MS analysis (semi-quantified) revealed pronounced shifts in the volatile profiles of all three rice groups during fermentation (Figure 3). Among the detected compound classes, esters showed the clearest accumulation, with low levels at the beginning of fermentation (day 0) followed by marked increases during fermentation (Figure 3A,C,E). Representative esters included ethyl 2-methylbutyrate, isoamyl acetate, ethyl hexanoate, ethyl lactate, and ethyl benzoate (Tables S2–S4). Organic acids, including acetic acid, isobutyric acid, hexanoic acid, and nonanoic acid (Tables S2–S4), also exhibited stage-dependent changes, generally increasing during fermentation before changing more slowly at later stages. The magnitude and temporal patterns of these changes differed among GR, IR, and JR. Higher alcohols also changed markedly during fermentation, with accumulation occurring mainly during the early-to-middle stages (days 2 to 14) and reaching a plateau in the late stage (day 35). Aldehydes and ketones also changed over time. Heterocyclic and other minor compounds such as pyrazines and furans exhibited more complex trajectories.

Principal component analysis separated samples primarily according to fermentation stage within each rice group (Figure 3B,D,F). The trajectories were broadly similar among GR, IR, and JR, but their positions and compound-class abundances differed. Variable importance in projection (VIP) was used to identify the most statistically significant variables. As shown in Figure S2A,C,E, the cumulative contribution rates of the PLS-DA models for the three fermented grain samples were 93.6%, 95.8%, and 91.0%, respectively. Samples from different stages were also roughly divided into three categories, consistent with the PCA results. In addition, permutation tests (*n* = 200) were performed on the models to assess whether the discriminant models overfitted the data. As shown in Figure S2B,D,F, all values on the left were lower than the original points on the right, and the regression line of the *Q*^2^ points had a negative intercept on the vertical axis, indicating that the model was not overfitted and had good predictive ability, and could be used for discriminant analysis of fermented grain samples. VIP was used to quantitatively analyze the contribution of aroma compounds in fermented grains. As shown in Figure S1, 18 flavor compounds with VIP > 1 were identified in both GR and IR fermented grains, while JR fermented grains contained 30 such compounds. Among them, 8 flavor compounds were shared among all three groups, namely (E)-2-octenal, benzaldehyde, phenylacetaldehyde, ethyl hexanoate, acetoin, ethyl 2-methylbutyrate, (E)-2-heptenal, and ethyl nicotinate. Notably, these volatile compounds were measured from fermented grains, and their abundances do not equate to the sensory properties of distilled baijiu.

### 3.4. Analysis of Microbial Diversity in Fermented Grains

Good’s coverage values ranged from 0.9944 to 0.9997 across all samples (Table 1). As shown in Table 1, α-diversity patterns varied with fermentation stage and rice substrate, and the trajectories differed between bacterial and fungal communities. In GR, bacterial Chao1 and Shannon indices increased throughout fermentation and reached their highest values at day 35 (Chao1 = 1514.73, Shannon = 6.25). The fungal Chao1 and Shannon indices in GR also gradually increased during fermentation and peaked at day 35 (Chao1 = 103.55, Shannon = 2.04), although the Shannon index showed a transient decrease at day 2. The bacterial Chao1 and Shannon indices in IR showed an increasing trend and reached their maximum values at day 35 (Chao1 = 850.43, Shannon = 2.70). Similarly, the fungal Chao1 and Shannon indices in IR gradually increased and peaked at day 35 (Chao1 = 134.98, Shannon = 2.32). A different pattern was observed in JR: the bacterial Chao1 and Shannon indices in JR peaked at day 14 (Chao1 = 742.79, Shannon = 2.78) and then declined sharply by day 35 (Chao1 = 329.59, Shannon = 1.73). For fungi in JR, the Chao1 richness index reached its maximum at day 14 (114.83) and subsequently decreased by day 35, whereas the Shannon diversity index continued to increase and reached 2.16 at day 35. Overall, these results demonstrate rice associated differences in the temporal development of within-sample bacterial and fungal diversity.

### 3.5. Microbial Succession During Fermentation

**Figure 4:**
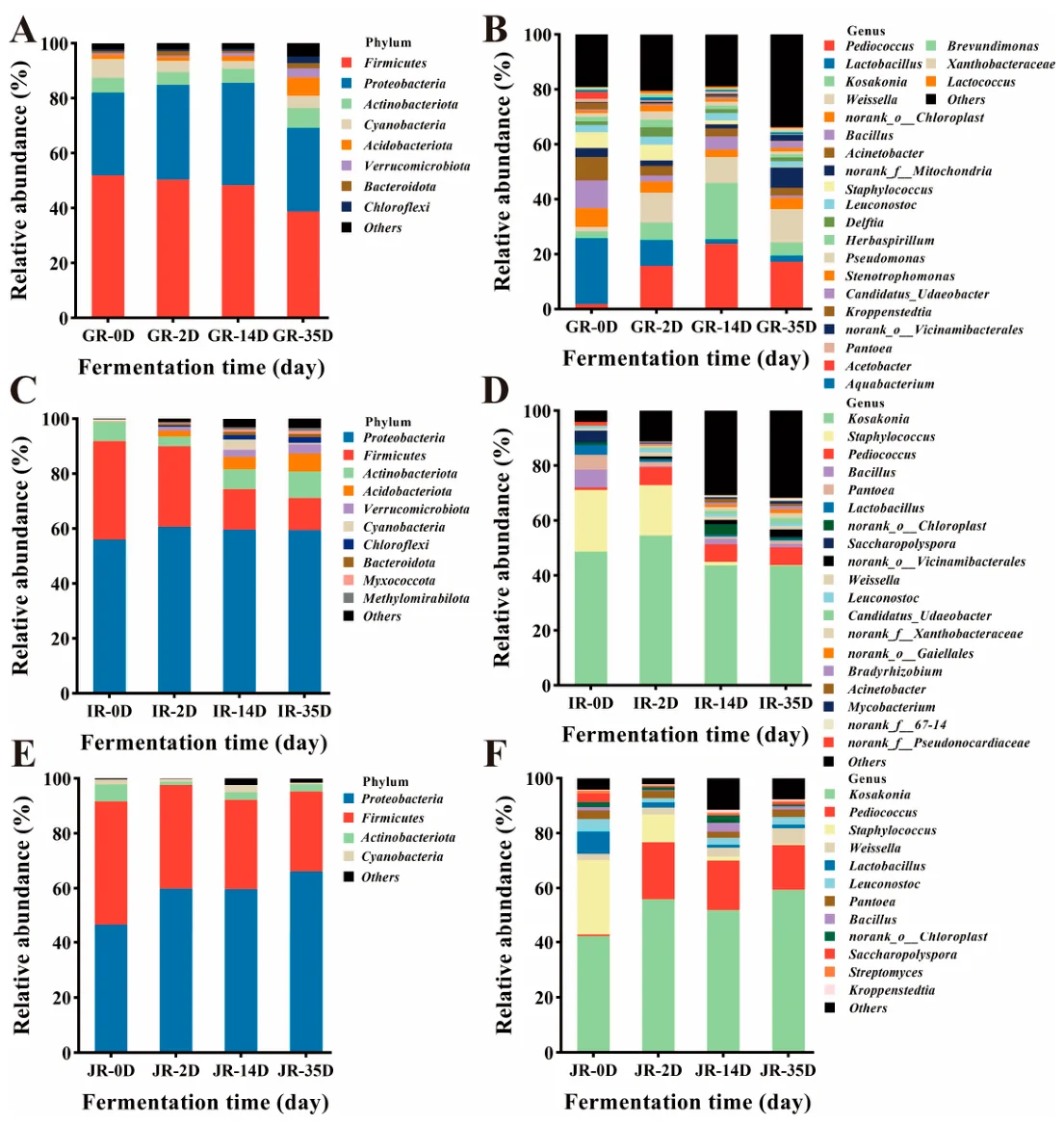
Temporal succession of bacterial communities in fermented grains prepared from three rice substrates. Panels (**A**,**C**,**E**) show phylum-level relative abundances in glutinous-rice (GR), indica-rice (IR), and japonica-rice (JR) fermentations, respectively; panels (**B**,**D**,**F**) show the corresponding genus-level relative abundances. Samples were collected at 0, 2, 14, and 35 days of fermentation.

As shown in Figure 4, bacterial communities underwent pronounced temporal restructuring during fermentation, with both shared and rice associated patterns. At the phylum level (Figure 4A,C,E), *Firmicutes* and *Proteobacteria* were the dominant phyla across all fermented grains. Their relative proportions varied among the three rice groups, with *Firmicutes* being dominant in GR, whereas *Proteobacteria* generally accounted for larger proportions in IR and JR. *Actinobacteriota* was also detected. At the genus level of bacterial communities (Figure 4B,D,F), a multi genus co-existence structure during the initial fermentation stage was observed. In GR at day 0, *Lactobacillus*, *Bacillus*, *Acinetobacter*, and *Staphylococcus* were among the dominant genera, while *Pediococcus*, *Kosakonia*, and *Weissella* gradually became more prominent as fermentation progressed. IR and JR showed more similar genus level profiles, with *Kosakonia* maintaining dominance throughout the fermentation stage. *Staphylococcus* was relatively abundant at 0–2 days but declined during middle and late fermentation. *Pediococcus* increased from day 2 and remained abundant through day 35.

**Figure 5:**
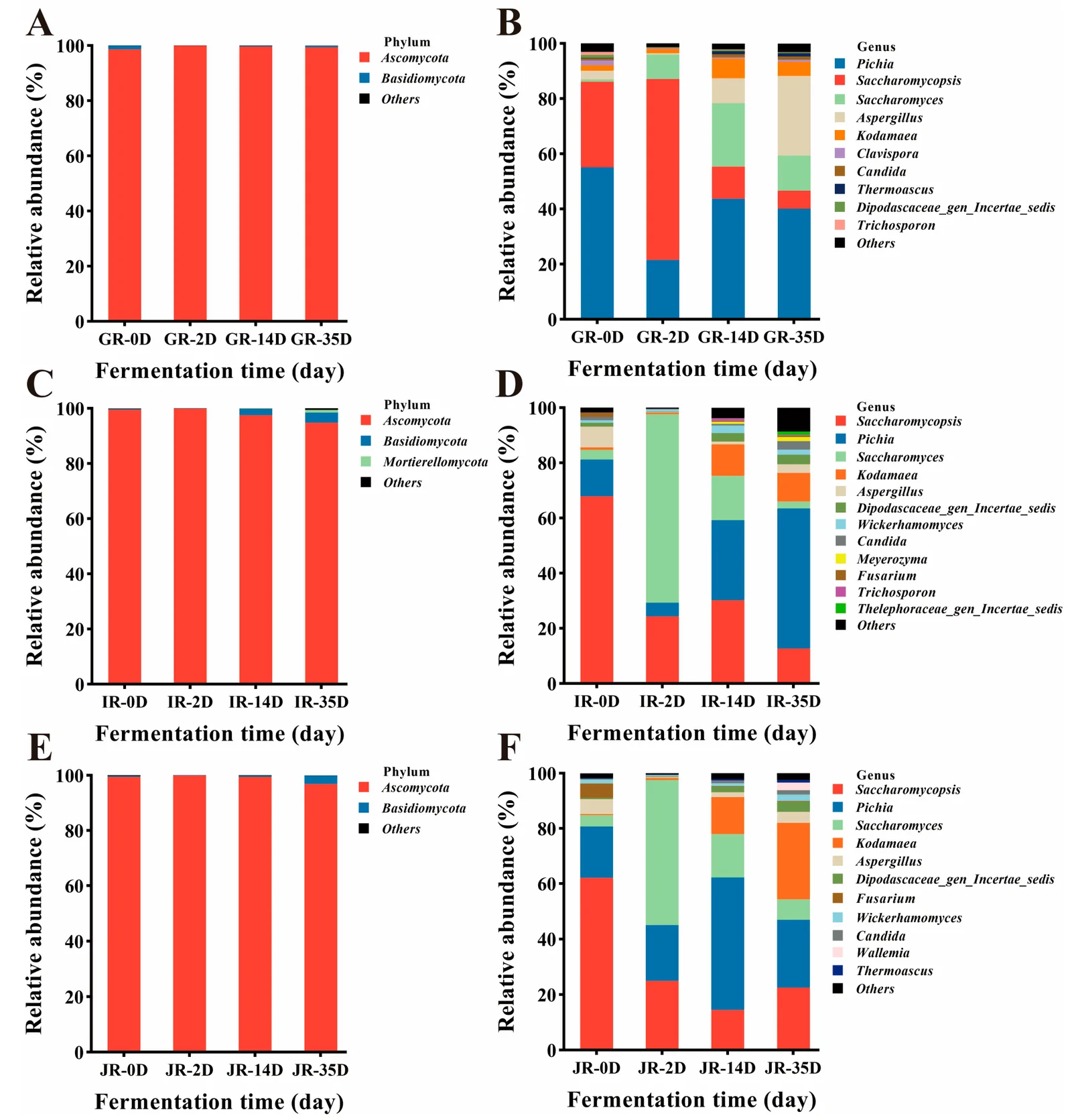
Temporal succession of fungal communities in fermented grains prepared from three rice substrates. Panels (**A**,**C**,**E**) show phylum-level relative abundances in glutinous-rice (GR), indica-rice (IR), and japonica-rice (JR) fermentations, respectively; panels (**B**,**D**,**F**) show the corresponding genus-level relative abundances. Samples were collected at 0, 2, 14, and 35 days of fermentation.

The fungal communities were dominated by Ascomycota throughout fermentation, accounting for more than 90% of the sequences in all three groups (Figure 5A,C,E). *Basidiomycota* and other phyla occurred at much lower relative abundances. At the genus level of fungal communities (Figure 5B,D,F), succession differed among the rice groups. At the beginning of fermentation, *Pichia* and *Saccharomycopsis* were dominant fungal genera, with *Pichia* being particularly abundant in GR, while *Saccharomycopsis* was more prominent in IR and JR. The relative abundance of *Saccharomyces* initially increased and then decreased with fermentation period, with its highest relative abundance in IR and JR observed on day 2. In contrast, *Aspergillus* and *Kodamaea* became more prominent during the later stages, with *Aspergillus* showing its highest relative abundance in GR at day 35 and *Kodamaea* being more strongly represented in late-stage IR and JR samples. Taken together, the coexistence of a broadly conserved phylum level framework with pronounced genus level turnover was observed across the three rice fermentations.

### 3.6. Associations Between Physicochemical Variables and Microbial Communities

**Figure 6:**
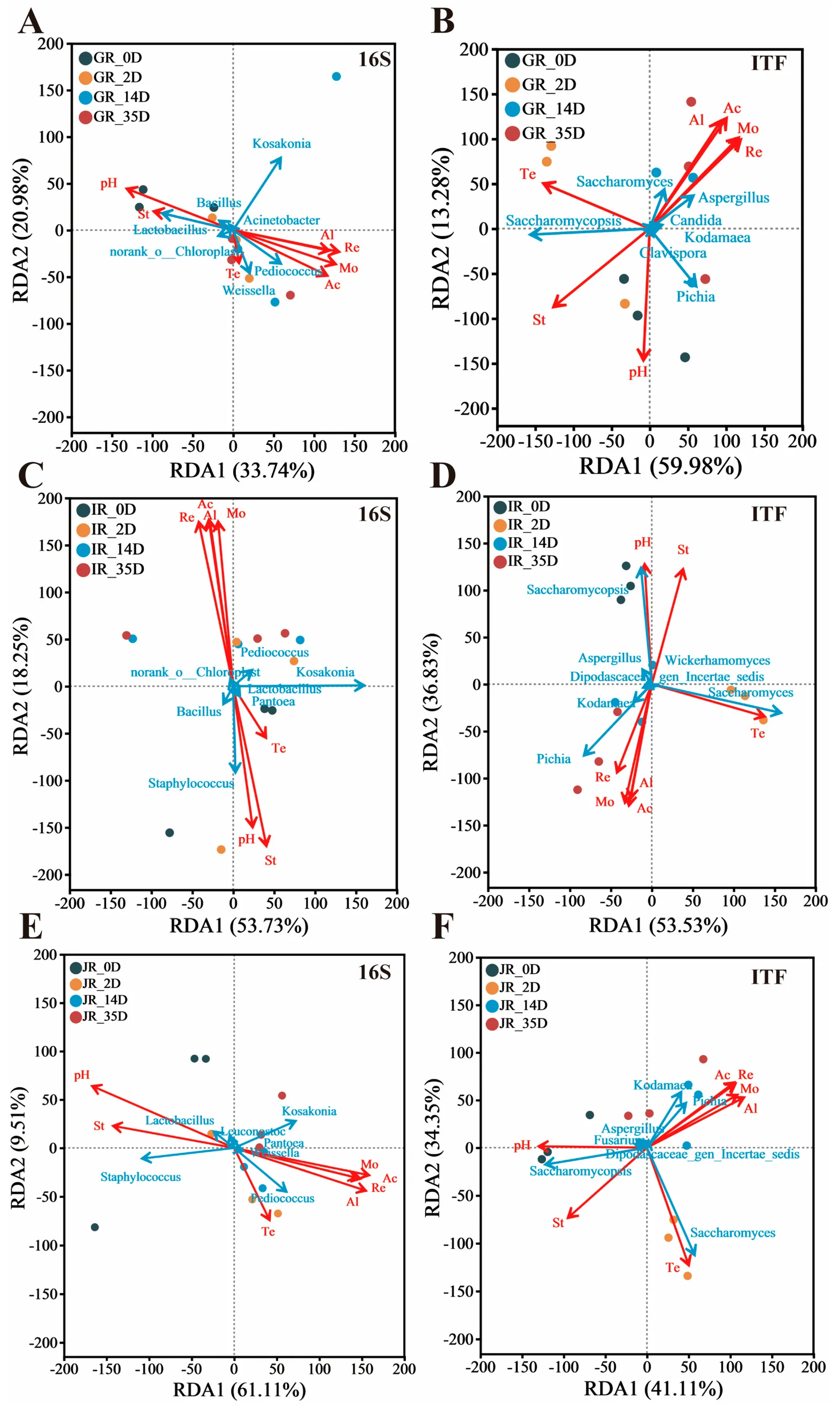
Redundancy analysis (RDA) of associations between physicochemical variables and microbial-community composition during fermentation. Panels (**A**,**C**,**E**) show bacterial communities in glutinous-rice (GR), indica-rice (IR), and japonica-rice (JR) at 0 (0D), 2 (2D), 14 (14D), and 35 (35D) days of fermentation, respectively; panels (**B**,**D**,**F**) show the corresponding fungal communities. Symbols represent samples collected at different fermentation stages; blue vectors represent selected microbial genera, and red vectors represent physicochemical variables. Te, temperature; Mo, moisture; Ac, titratable acidity; St, starch; Re, reducing sugars; Al, alcohol content.

Redundancy analysis (RDA) was employed to investigate associations between the physicochemical trajectories and microbial community succession in the three rice fermentations (Figure 6). For bacterial communities (Figure 6A,C,E), the first two constrained axes captured 54.72%, 71.98%, and 70.62% of the variation represented by the RDA ordinations for GR, IR, and JR, respectively. The corresponding proportions for fungal communities (Figure 6B,D,F) were 73.26%, 90.36%, and 75.46%.

Across the three rice groups, pH and starch content most strongly aligned with early-stage samples (0–2 days), which contained relatively high abundances of taxa including bacteria such as *Lactobacillus* and *Staphylococcus*, and fungi such as *Saccharomycopsis* and *Pichia* (Figure 6). In contrast, temperature, moisture, acidity, reducing sugar, and alcohol content were more closely oriented toward the middle and late-stage samples (14–35 days), which may be related to the succession to a mature community dominated by *Kosakonia*, *Pantoea*, *Pediococcus*, *Kodamaea*, *Saccharomyces* and *Aspergillus*. Notably, the taxa associated with these physicochemical gradients differed among GR, IR, and JR.

### 3.7. Associations Among Microbial Taxa and Volatile Flavor Compounds

**Figure 7:**
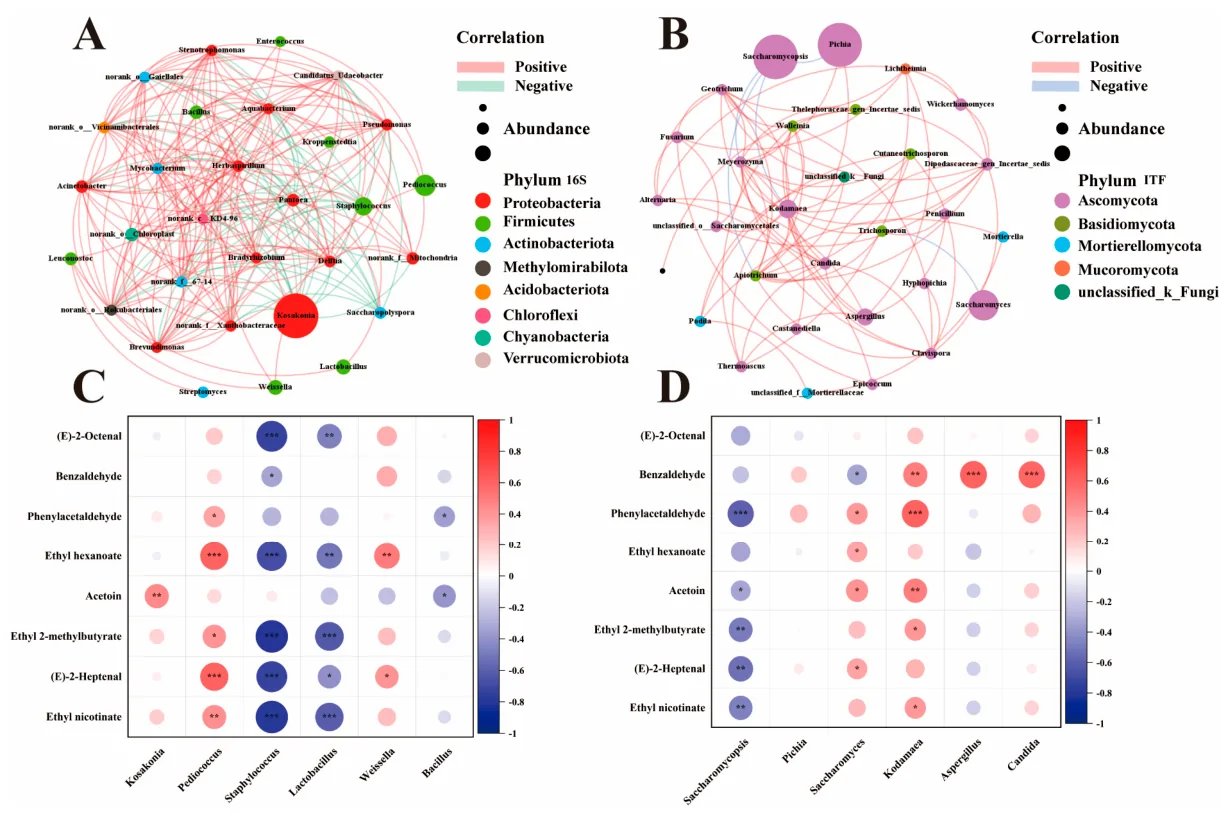
Microbial co-occurrence patterns and associations between microbial genera and selected volatile compounds during fermentation. (**A**) Bacterial co-occurrence network; (**B**) fungal co-occurrence network; (**C**) associations between selected bacterial genera and volatile compounds; and (**D**) associations between selected fungal genera and volatile compounds. * *p* < 0.05, ** *p* < 0.01, *** *p* < 0.001.

In the bacterial co-occurrence network (Figure 7A), *Kosakonia* showed high degree and betweenness centrality in network topology, identifying it as a potential keystone node based on network statistics. However, its correlations with the volatile compounds selected from Section 3.3 were comparatively weak (Figure 7C). In contrast, functional bacteria such as *Pediococcus* and *Lactobacillus* showed strong associations with flavor formation. Specifically, *Pediococcus* was positively correlated with several ester and aldehyde related flavor substances, including ethyl hexanoate and (E)-2-heptenal, while *Lactobacillus* exerted negative correlations with ethyl-2-methylbutyrate and ethyl nicotinate. *Staphylococcus* also showed predominantly negative associations with several selected aldehyde and ester compounds.

A comparable pattern was observed for fungi (Figure 7B). *Pichia* was a prominent fungal node, while *Saccharomycopsis*, *Kodamaea*, and *Saccharomyces* showed stronger associations with selected volatile compounds (Figure 7D). *Kodamaea* was positively associated with phenylacetaldehyde and various esters. *Saccharomyces* was positively correlated with multiple core flavor compounds including phenylacetaldehyde, ethyl hexanoate, acetoin, and (E)-2-heptenal. *Saccharomycopsis* exerted negative associations with several of the same variables, including phenylacetaldehyde, ethyl 2-methylbutyrate, (E)-2-heptenal, and ethyl nicotinate. Collectively, these results indicate that the taxa most prominent within microbial-community networks were not necessarily those most strongly associated with volatile-metabolite variation. Rather, distinct bacterial and fungal genera showed compound-specific positive or negative covariation during fermentation.

## 4. Discussion

The dynamic changes in physicochemical factors within fermented grains are directly linked to the growth and metabolism of microorganisms, as well as the formation of various flavor compounds [14]. In the present study, the coordinated early increases in temperature and moisture are consistent with intense early microbial activity in the initial stage of solid-state fermentation, which can generate heat and metabolic water during substrate (e.g., glucose) conversion [15]. The sharp pH decline within the first 7 days is more plausibly interpreted as the combined consequence of organic acid accumulation and dissolved CO_2_ produced by aerobic respiration in the early stage [16]. The non-monotonic pattern of titratable acidity indicates that acid production, consumption, and conversion are dynamically balanced as the microbial community and chemical environment change, and reflects the buffering capacity of the fermentation matrix [17].

Changes in carbohydrate utilization were more directly indicative of fermentation progression. The inverse trend between starch decline and reducing sugar accumulation suggests that starch hydrolysis initially supplied fermentable sugars faster than they were consumed, whereas sugar utilization increasingly matched or exceeded sugar release as fermentation progressed [18]. The rapid early increase in alcohol content followed by a plateau reflects progressive conversion of fermentable sugars to ethanol followed by a decline in net ethanol production as readily available substrates decreased and inhibitory metabolites accumulated [19]. Similar raw material dependent differences in carbohydrate utilization and physicochemical trajectories have been reported during light-flavor baijiu fermentation using different sorghum substrates [13,20].

Traditional sensory evaluation of fermented grains relies heavily on subjective human judgment, which often suffers from poor reproducibility. In contrast, the electronic nose, an intelligent instrumental analogue of the human olfactory system, provides an objective and standardized means of odor profiling, effectively circumventing inter-operator variability [21]. In the present study, electronic-nose analysis captured progressive shifts in the overall odor fingerprint of fermented grains throughout fermentation. These sensor outputs reflect integrated bulk olfactory signals and cannot be used to identify specific classes of volatile compounds.

GC-MS analysis provided a comprehensive volatile profile of the fermented grains, which are central to the final flavor characteristics of the baijiu. Esters were the most important aroma compounds in baijiu, the esters accumulating in fermented grains serve as key precursors that may produce fruity, floral, and sweet notes after distillation [1]. The pronounced late-stage ester accumulation observed in the present rice fermentations differs from the early ester maximum reported in some sorghum-based light-flavor baijiu systems, emphasizing that volatile trajectories are strongly process and substrate-dependent [20]. The magnitude and temporal patterns of organic acid changes differed among GR, IR, and JR, indicating rice associated variation in volatile metabolite development [6].

Higher alcohols contribute to the body and flavor of baijiu, while also introducing undesirable sensory notes when excessive [22]. The accumulation of alcohols mainly during the early to middle stages is compatible with yeast mediated amino acid catabolism through routes such as the Ehrlich pathway in the initial process, which can generate alcohols like isoamyl alcohol, isobutanol, and phenethyl alcohol [23], while oxygen depletion and rising ethanol concentrations in the late stage jointly inhibited yeast metabolism, leading to a stabilized alcohol level. Aldehydes and ketones also changed over time, consistent with their roles as intermediates or products of microbial metabolism. Heterocyclic and other minor compounds such as pyrazines and furans exhibited more complex trajectories and may reflect contributions from thermally generated precursors (Maillard products) in the raw materials or Daqu together with subsequent microbial transformation [24].

The different responses of richness and Shannon diversity indicate that changes in the number of detected taxa and their relative-abundance distribution were not always synchronous. The initial rice starch properties, particularly the amylose and amylopectin contents (Table S1), may be one set of contributing factors that could shape microbial community diversity by modulating the accessibility and fermentation kinetics of the carbon source. Starch with a higher proportion of amylopectin (GR) is generally more readily fermented, the bacterial and fungal Chao1 and Shannon indices increased with fermentation time, and the high amylopectin content could continuously supply available carbon sources for microorganisms, promoting species enrichment and enhancing community diversity [6]. The community diversity of IR also increased during fermentation, but the bacterial Shannon index was overall lower than that of glutinous rice, which may be related to the inhibition of some microbial growth by high tannins in the early stage; community diversity gradually recovered as tannins degraded [25]. The bacterial alpha diversity of JR first increased and then decreased, which may be related to the stress imposed on bacteria by nutrient depletion and accumulation of acidic metabolites in the late fermentation stage [26]. These differences indicate that richness and diversity reflect the balance between persistence and dominance under a specific substrate and process environment, rather than a universal direction in all baijiu fermentation processes.

At the phylum level, *Firmicutes* and *Proteobacteria* were the dominant phyla across all fermented grains, consistent with the core microbial community structure of traditional rice based fermented grains [17], and fermentation of soy sauce flavor, sesame flavor, and strong flavor baijiu [27,28,29]. *Actinobacteriota* could contribute hydrolytic enzymes (e.g., *amylases*, *proteases*, *and lipases*), thereby efficiently decomposing macromolecular substances into compounds such as alcohols and fatty acid esters, further enhancing the flavor of distilled spirits [30]. At the genus level, lactic acid bacteria such as *Lactobacillus* and *Pediococcus* can influence acid production (e.g., lactic acid and acetic acid) and provide metabolites or precursors that participate in flavor formation during baijiu fermentation [31]. *Kosakonia* is also the core bacterial genus in traditionally fermented cereal sour congee in China [32]. The enrichment of *Staphylococcus* in the early fermentation stage may play an important role in organic acid metabolism, as reported in strong flavor fermented grains [33]. The persistence of *Pediococcus* during the middle and late stages is biologically plausible in the increasingly stressful fermentation environment, as baijiu derived *Pediococcus*, *pentosaceus* demonstrated coordinated responses to lactic acid and ethanol stress. This bacterial genus could also synergistically interact with yeast to participate in the formation of organic acids, alcohols, and esters, influencing the flavor and quality of the base liquor [34].

For fungal communities, *Ascomycota* represented the predominant core fungal phylum related to the fermentation process. Previous studies have associated Pichia with ester formation, including ethyl acetate production, thereby imparting floral and fruity notes to the liquor [35]. Although *Saccharomycopsis* is not a primary fermentation yeast, it contributes to the secretion of starch degrading enzymes that can influence substrate utilization and aroma development [36]. The early peak of *Saccharomyces* is consistent with an early role in glucose conversion and ethanol formation via the glycolytic pathway [37]. The late persistence of *Aspergillus* and *Kodamaea* suggests adaptation to the increasingly acidic, nutrient limited, and alcohol containing fermentation environment [38]. The coexistence of a broadly conserved phylum level framework with pronounced genus level turnover suggests that rice substrate variation did not fundamentally replace the major microbial lineages participating in fermentation, but rather altered the relative success of taxa within these lineages. This pattern is consistent with recent raw material comparison studies in light-flavor baijiu, in which differences among grain substrates were expressed particularly strongly at lower taxonomic levels and, in some cases, were more pronounced in fungal than bacterial communities [13,20].

RDA results indicated that the measured physicochemical variables were closely associated with the major gradients in bacterial and fungal community composition [17]. Similar coupling between carbohydrate related variables and microbial community restructuring has been observed in rice flavor baijiu produced from indica and japonica rice [17,20]. These associations are biologically plausible because physicochemical changes may jointly reshape the fermentation niche by modifying carbon availability, substrate hydration, and acid ethanol stress. The resulting environmental filtering could favor stress tolerant taxa, which may partly explain the increased relative abundance of Pediococcus during the later stages of fermentation [34]. Correlation analyses in Tuqu-type light-flavor baijiu fermented grains also confirmed that moisture and acidity were tightly linked to the succession of lactic acid bacteria and yeast populations throughout fermentation [39]. Notably, microbe volatile relationships in solid state fermentation may be bidirectional and embedded within broader community interactions. Previous work in baijiu starter ecosystems has shown that microbial volatile organic compounds can themselves modify microbial interactions [40]. Overall, these results reveal a potential successional trajectory, where pH and starch availability were associated with microbial dynamics in the initial fermentation stage, while temperature, moisture, and metabolic end products (acidity, alcohol) were associated with community shifts in the later stage. Moreover, variations in initial abiotic parameters could alter the deterministic-stochastic balance of microbial community assembly and reshape the final volatile profile of light-flavor baijiu fermented grains [41]. These factors are linked to the synergistic succession of bacterial and fungal communities and lay the foundation for the functional maturation of the fermented grains.

In the bacterial co-occurrence network, *Kosakonia* displayed topological features suggestive of a potential keystone node, but its correlations with the selected volatile compounds were comparatively weak. Indicating that its core function relates to community structure rather than being closely associated with flavor synthesis [33]. In contrast, functional bacteria such as *Pediococcus* and *Lactobacillus* showed strong associations with flavor formation. Similar positive associations between *Pediococcus* and ethyl hexanoate have been reported in other baijiu fermentation systems [42,43]. These associations may reflect the contribution of *Pediococcus* to an acid and precursor rich microbial environment favorable for ester accumulation. *Staphylococcus* also showed predominantly negative associations with several selected aldehyde and ester compounds [42,43]. For fungi, *Kodamaea* was positively associated with phenylacetaldehyde and various esters, suggesting a possible linkage to floral and fruity flavor profiles. *Saccharomyces* was positively correlated with multiple core flavor compounds including phenylacetaldehyde, ethyl hexanoate, acetoin, and (E)-2-heptenal. This is in line with previous reports that *S. cerevisiae* strains have enhanced ethyl-hexanoate-producing capacity and can alter ethyl hexanoate formation in baijiu fermentation [43,44]. *Saccharomycopsis* showed negative associations with several of the same variables, which agrees with a previous study reporting that experimental fortification of light-flavor baijiu with *Saccharomycopsis fibuligera* significantly affects ethyl acetate and restructures the fungal community [4]. Collectively, these results indicate that the taxa most prominent within microbial community networks were not necessarily those most strongly associated with volatile metabolite variation. Rather, distinct bacterial and fungal genera showed compound specific positive or negative covariation during fermentation. Notably, correlations between microbial taxa and flavor compounds only reflect statistical associations rather than causality. Physicochemical covariation and microbial interactions in fermented grains may confound these links. These statistical covariations cannot confirm direct synergistic or antagonistic biological interactions but hint that microbial community dynamics may collectively shape the final flavor characteristics of light-flavor baijiu. Comparable microbe metabolite associations have been reported during light-flavor baijiu fermentation [35].

Beyond these ecological associations, rice varietal differences bear practical implications for baijiu manufacturing. Rice genotype modulates substrate properties, microbial succession, and volatile accumulation in fermented grains. Selecting suitable rice varieties helps regulate fermentation performance and flavor precursor formation, and raw material switching may require parameter adjustment to stabilize fermentation and flavor. Although obtained from pilot-scale trials, these findings support raw material screening and formula optimization for rice-based light-flavor baijiu. Nevertheless, only three cultivars were tested. While representative for rice-based light-flavor baijiu fermentation, these results cannot be generalized to all rice genotypes. Future work with more rice varieties of diverse grain traits is needed to validate our observations and establish broader links between rice properties, microbial succession and flavor precursor formation.

## 5. Conclusions

This study systematically revealed the mechanism by which different rice varieties influenced the flavor of light-flavor baijiu through regulating the microecological environment, microbial succession and volatile-metabolite development during light-flavor baijiu fermentation. The three rice materials exhibited distinct physicochemical trajectories and observable genus-level microbial successional patterns, while sharing a bacterial community dominated by *Firmicutes* and *Proteobacteria* and a fungal community dominated by *Ascomycota*. At the genus level, *Lactobacillus*, *Pediococcus*, *Pichia*, and *Saccharomyces* exhibited clear raw material specificity, showing strong associations with community succession. pH and starch content were linked more strongly with early fermentation, while temperature, moisture, acidity, and alcohol content were associated with changes in later stages. Flavor compound metabolism was highly synchronized with microbial succession, with key volatile compounds such as esters, aldehydes and ketones exhibiting stage-specific accumulation. The co-occurrence network further indicated that microbial communities were linked to flavor characteristics through synergistic and antagonistic interactions. However, the present volatile data characterize fermentation-stage flavor potential rather than the final sensory quality of distilled baijiu. How these fermented-grain metabolites transfer and transform during distillation to shape liquor sensory properties requires further investigation. These findings provide a process-level perspective on raw-material-associated variation in rice-based light-flavor baijiu fermentation and support the rational selection of rice materials and optimization of fermentation strategies.

## Figures and Tables

**Table 1:** Alpha-diversity metrics of bacterial and fungal communities during fermentation of three rice substrates.

Samples			Chao1	Shannon	Coverage	Samples			Chao1	Shannon	Coverage
Bacteria	GR	0D	354.24 ± 49.25 ^d^	3.75 ± 0.17 ^c^	0.9991	Fungi	GR	0D	89.01 ± 7.6 ^b^	1.45 ± 0.02 ^b^	0.9996
		2D	485.69 ± 35.7 ^c^	4.15 ± 0.29 ^bc^	0.9990			2D	92 ± 4.78 ^ab^	0.98 ± 0.18 ^c^	0.9995
		14D	669.57 ± 31.01 ^b^	4.43 ± 0.23^b^	0.9989			14D	99.9 ± 0.8 ^ab^	1.79 ± 0.09 ^a^	0.9996
		35D	1514.73 ± 47.35 ^a^	6.25 ± 0.09 ^a^	0.9974			35D	103.55 ± 4.91 ^a^	2.04 ± 0.12 ^a^	0.9996
	IR	0D	303.65 ± 5.54 ^c^	1.26 ± 0.15 ^d^	0.9986		IR	0D	91.27 ± 2.01 ^b^	1.34 ± 0.05 ^c^	0.9996
		2D	336.97 ± 11.71 ^c^	1.64 ± 0.04 ^c^	0.9944			2D	91.06 ± 1.01 ^b^	1.17 ± 0.07 ^d^	0.9994
		14D	550.41 ± 21.12 ^b^	1.96 ± 0.04 ^b^	0.9977			14D	126.66 ± 4.02 ^a^	2.06 ± 0.05 ^b^	0.9996
		35D	850.43 ± 30.03 ^a^	2.7 ± 0.08 ^a^	0.9949			35D	134.98 ± 4.47 ^a^	2.32 ± 0.08 ^a^	0.9995
	JR	0D	251.51 ± 31.22 ^c^	1.96 ± 0.07 ^b^	0.9982		JR	0D	105.18 ± 2.92 ^b^	1.39 ± 0.07 ^c^	0.9995
		2D	340.17 ± 18.32 ^b^	1.62 ± 0.07 ^b^	0.9986			2D	112.20 ± 4.31 ^ab^	1.30 ± 0.03 ^c^	0.9994
		14D	742.79 ± 31.59 ^a^	2.78 ± 0.07 ^a^	0.9969			14D	114.83 ± 3.95 ^a^	1.69 ± 0.16 ^b^	0.9995
		35D	329.59 ± 11.37 ^b^	1.73 ± 0.4 ^b^	0.9981			35D	94.05 ± 3.21 ^c^	2.16 ± 0.03 ^a^	0.9997

Note: Values are presented as mean ± SD (n = 3). Chao1 represents estimated richness, Shannon represents community diversity, and Good’s coverage indicates sequencing completeness. Different lowercase superscript letters within the same rice group indicate significant differences among fermentation stages (*p* < 0.05, one-way ANOVA followed by Tukey post-hoc test). GR, glutinous rice; IR, indica rice; JR, japonica rice; D, fermentation day.

## Data Availability

The original contributions presented in this study are included in the article/Supplementary Materials. Further inquiries can be directed to the corresponding author.

## Supplementary Materials

The following supporting information can be downloaded at: https://www.mdpi.com/article/10.3390/foods15213825/s1, Text S1. Analytical methods for physicochemical characteristics of raw rice materials; Table S1: Physicochemical characteristics of the three rice materials used for fermentation; Table S2: Semi-quantitative levels of volatile compounds detected in glutinous-rice fermented grains (GR) during fermentation; Table S3: Semi-quantitative levels of volatile compounds detected in indica-rice fermented grains (IR) during fermentation. Table S4: Semi-quantitative levels of volatile compounds detected in japonica-rice fermented grains (JR) during fermentation. Figure S1: Variable importance in projection (VIP) scores of semi-quantitative levels of volatile compounds contributing to discrimination among fermentation stages in (A) glutinous-rice (GR), (B) indica-rice (IR), and (C) japonica-rice (JR) fermentations, derived from PLS-DA models. The red bar indicates the compound with VIP > 1. Figure S2: PLS-DA model analysis and permutation test (n = 200) of volatile flavor compounds in fermented grains. Panels (A,C,E) show the PLS-DA score plots based on the complete volatile profiles within each rice group; panels (B,D,F) show the permutation tests.
