# Supplementary material for: Physicochemical Dynamics, Microbial Succession, and Volatile Profiles of Light-Flavor Baijiu Fermented Grains Prepared from Different Rice Varieties
Source: Foods. 2026 Oct 26;15(21):3825. doi: 10.3390/foods15213825 (PMC13763087; doi:10.3390/foods15213825)
Supplement: Supplementary file 1 [file foods-15-03825-s001.zip › foods-4607511-supplementary.pdf]

Physicochemical dynamics, microbial succession, and volatile profiles of light-flavor baijiu fermented grains prepared from different rice varieties

Wenjun Rao, Xin Li, Yaoyao Qi, Shuai Hu, Linghua Ouyang, Shuilan Zhu, Shuhan Yi, Jinying Zhou*

Institute of Agricultural Products Processing, Jiangxi Academy of Agricultural Sciences, Nanchang, Jiangxi 330200, China

*Corresponding author: Jinying Zhou; 602 Nanlian Road, Institute of Agricultural Products Processing, Jiangxi Academy of Agricultural Sciences, Nanchang, Jiangxi, 330200, China; E-mail: zhou_jiny@163.com; Mobile: +86-13677914955

Table S1. Physicochemical characteristics of the three rice materials used for fermentation.

	GR	IR	JR
Starch (g/100g)	75.06 ± 1.24^{a}	74.07 ± 4.91^{a}	77.44 ± 3.87^{a}
Amylose (g/100g)	2.69 ± 0.36^{b}	11.18 ± 0.06^{a}	11.75 ± 0.56^{a}
Amylopectin (g/100g)	46.64 ± 2.18^{a}	29.39 ± 2.53^{c}	36.82 ± 0.32^{b}
Protein (g/100g)	7.63 ± 0.57^{a}	8.73 ± 0.95^{a}	8.61 ± 0.57^{a}
Crude fat (%)	1.34 ± 0.18^{a}	1.58 ± 0.12^{a}	0.62 ± 0.09^{b}
Ash (g/100g)	1.21 ± 0.06^{a}	1.13 ± 0.10^{ab}	1.01 ± 0.03^{b}
Tannin (%)	0.41 ± 0.00^{b}	1.52 ± 0.05^{a}	0.41 ± 0.00^{b}

Note: Values are presented as mean ± SD (n = 3). GR, glutinous rice; IR, indica rice; JR, japonica rice. Different lowercase letters within the same row indicate significant differences among the three rice materials ($p < 0.05$, one-way ANOVA followed by Tukey post-hoc test). Total starch was measured by acid-hydrolysis-DNS method (GB 5009.9-2016). Amylose and amylopectin were determined via dual-wavelength iodine colorimetry (T/QAS 076-2022). Partial resistant starch is not fully captured by the dual-wavelength colorimetric assay, which explains why the sum of amylose and amylopectin is lower than the total-starch value.

Text S1. Analytical methods for physicochemical characteristics of raw rice materials

All compositional analyses of rice raw materials were performed on a dry-weight basis. Crude protein was determined by the Kjeldahl method, following Chinese

National Standard GB 5009.5-2016. An automatic Kjeldahl nitrogen analyzer (Model ATN-300, Chongqing Drawell Instrument Co., Ltd., China) was used for the measurement. Total starch content was quantified by acid hydrolysis-DNS method according to Chinese National Standard GB 5009.9-2016. A microplate reader (Model FlexA-200) was applied for color detection. Ash content was determined by direct ashing method in accordance with Chinese National Standard GB 5009.4-2016. A 0.1 mg analytical balance (Model ME54, METTLER TOLEDO, Switzerland) was used for gravimetric weighing. Crude fat was measured by Soxhlet extraction following Chinese National Standard GB 5009.6-2016 with a Soxhlet extractor. Amylose and amylopectin contents were determined by dual-wavelength colorimetric method according to T/QAS 076-2022 using an ultraviolet-visible spectrophotometer (Model UV 8000, Shanghai Yoke Instrument Co., Ltd., China). Tannin content was determined via dimethylformamide extraction and colorimetric method following Chinese National Standard GB/T 15686-2008. The UV 8000 ultraviolet-visible spectrophotometer was used for absorbance measurement. All assays were performed with three biological replicates for each rice variety.

Table S2. Semi-quantitative levels of volatile compounds detected in glutinous-rice fermented grains (GR) during fermentation.

No.	Aroma compounds	Concentration (μg/g)			
		GR-0D	GR-2D	GR-14D	GR-35D
1	Isobutyl acetate	ND	ND	189.8±18.32	214.82±37.53
2	Ethyl butanoate	ND	364.94±10.52	864.54±40.53	876.74±71.51
3	Ethyl 2-methylbutyrate	ND	58.75±2.96	728.47±45.93	1384±95.47
4	Ethyl 3-methylbutanoate	ND	ND	132.28±13.32	239.31±9.5
5	Isoamyl acetate	ND	246.79±36.09	617.53±147.69	701.89±69.14
6	Ethyl valerate	ND	ND	945.93±1.46	988.72±73.36
7	Ethyl hexanoate	14.52±0.24	1127.13±37.11	4132.5±326.81	4816.48±487.83
8	Ethyl heptanoate	ND	126.88±10.77	110.03±5.99	105.81±4.37
9	Ethyl lactate	ND	ND	952.43±1232.44	2440.15±1803.26
10	Octyl acetate	25.85±1.68	21.59±1.42	25.68±0.42	29.37±0.21
11	Ethyl nonanoate	2.62±0.58	25.58±4.83	63.86±7.86	69.09±3.59
12	Ethyl DL-Leucate	ND	19.81±1.33	83.9±2.92	117.39±2.31
13	Ethyl 3-(methylthio)propionate	ND	5.3±0.83	43.58±1.82	48.29±2.92
14	Diethyl malonate	ND	ND	ND	5.62±0.13
15	γ-Butyrolactone	ND	75.06±6.45	409.69±12.58	370.5±9.95
16	Ethyl decanoate	3.39±0.11	436.29±14.3	472.35±14.89	638.69±33.94
17	Isoamyl octanoate	ND	ND	24.09±0.08	27.69±0.73
18	Ethyl benzoate	45.22±3.55	144.53±1.11	624.83±21.6	1014.95±10.46
19	Ethyl 3-hydroxyhexanoate	ND	ND	ND	9.1±0.18
20	Linalyl butyrate	3.29±0.17	3.33±0.05	6.6±0.44	5.81±0.06
21	Diethyl succinate	1.43±0.02	76.15±2.98	ND	ND
22	Ethyl phenylacetate	ND	17.49±1.2	173.17±3.11	267.32±10.25
23	Ethyl nicotinate	ND	4.78±0.48	90.81±2.74	270±10.44
24	Ethyl laurate	0.19±0.03	13.48±2.09	15.74±0.42	12.65±1.66
25	γ-Nonalactone	0.91±0.03	2.68±0.13	5.77±0.4	6.94±0.15
26	Ethyl myristate	1.82±0.11	26.38±2.26	63.66±2.76	61.96±2.25
27	Ethyl stearate	0.03±0.01	4.67±0.14	10.89±0.53	10.34±0.26
28	Ethyl palmitate	6.61±0.62	ND	ND	ND
29	Acetic acid	ND	ND	4751.65±572.08	6755.39±434.37
30	Hexanoic acid	4.01±0.16	23.2±1.51	22.55±1.36	27.44±3.12
31	Isobutanol	25.15±2.08	36.42±1.57	67.26±3.77	88.86±14.72
32	1-Butanol	ND	ND	392.24±10.71	418.97±27.21
33	Isoamyl alcohol	309.48±9.78	ND	ND	ND
34	1-Pentanol	74.14±10.63	133.83±19.92	148.23±19.29	170.27±18.39
35	2-Heptanol	ND	36.04±1.09	56.08±1.43	62.71±1.85
36	1-Hexanol	1492.49±73.38	4178.83±177.81	4344.76±39.86	4493.14±67.38
37	3-Ethoxy-1-propanol	ND	189.03±20.01	274.13±4.45	311.83±7.28
38	3-Octanol	32.68±0.35	41.02±0.68	72.69±3.6	74.18±1.23
39	1-Heptanol	463.54±25.99	354.08±29.03	315.92±8.3	333.7±4.04
40	2-Ethylhexanol	22.34±1.62	19.95±0.3	23.55±2.05	26.01±0.7

41	2,3-Butanediol	ND	666.14±18.73	1915.74±24.12	2189.44±146.52
42	1-Octanol	72.75±2.31	241.95±31.56	236.47±17.82	252.18±10.34
43	1-Nonanol	52.53±1.15	84.31±1.57	94.86±2.38	85.67±3.86
44	1-Decanol	7.04±0.06	22.21±0.89	25.36±3.06	20.77±0.69
45	Guaiacol	0.1±0.01	1.11±0.1	0.38±0	0.4±0.01
46	Geraniol	ND	ND	1.36±0.27	1.17±0.15
47	Benzyl alcohol	1.11±0.01	1.24±0.02	1.29±0.05	1.48±0.02
48	Butylated hydroxytoluene	0.12±0.01	0.07±0	0.05±0	0.04±0
49	4-Ethyl-2-methoxyphenol	0.07±0.03	13.19±1.34	11.95±0.72	12.45±0.37
50	4-Ethylphenol	ND	10.98±0.74	6.48±0.56	6.87±0.23
51	3-Ethylphenol	ND	7.66±1.24	4.81±0.42	5.09±0.18
52	p-Vinylguaiacol	2.68±0.16	33.22±0.81	31.68±1.33	32.9±2.01
53	1-Tetradecanol	ND	1.41±0.08	2.43±0.11	2.41±0.1
54	4-Methoxybenzyl alcohol	ND	0.27±0.07	0.6±0.07	0.55±0.04
55	1-Hexadecanol	ND	0.28±0.06	0.52±0.01	0.46±0.02
56	α-Amylcinnamyl alcohol	ND	2.13±0.25	3.83±0.14	3.79±0.11
57	Stearyl alcohol	ND	0.19±0.01	0.48±0.01	0.48±0.01
58	Hexanal	128.53±18.5	78.17±8.34	128.25±49.64	198.08±14.46
59	Heptanal	28.69±1.53	27.88±1	39.68±4.13	41.6±4.92
60	(E)-2-Heptenal	330.66±26.27	691.92±55.04	799.87±164.38	1063.51±99.71
61	(E)-2-Octenal	ND	ND	4603.33±25.53	5262.5±90.8
62	Benzaldehyde	437.67±21.01	763.83±10.94	790±3.34	1308.77±17.81
63	Phenylacetaldehyde	ND	744.83±39.41	264.86±3.73	288.29±9.56
64	Pentadecanal	0.36±0.01	2.8±0.2	1.58±0.02	0.88±0.07
65	2-Heptanone	64.72±4.38	101.49±6.49	103.48±7.9	114.66±11.76
66	Acetoin	21.81±0.4	503.68±5.46	133.59±2.2	138.01±1.86
67	1-Octen-3-one	40.57±5.73	50.82±5.74	57.26±7.16	69.06±9.55
68	6-Methyl-5-hepten-2-one	33.02±8.63	69.06±13.14	73.3±11.49	70.82±3.93
69	2-Undecanone	ND	ND	ND	13.35±1.78
70	Pyridine	17.92±2.84	14.8±2.81	13.5±1.02	14.6±0.7
71	2-Acetylpyrrole	ND	0.13±0	0.18±0.02	0.19±0.01
72	2,5-Dimethylpyrazine	12.73±0.66	14.02±0.92	13.13±0.23	16.42±0.2
73	2,6-Dimethylpyrazine	ND	23.01±2.59	21.06±0.32	17.54±1.72
74	2,3-Dimethylpyrazine	12.14±3.4	9.23±0.87	9.03±1.27	9.72±0.13
75	2-Ethyl-5-methylpyrazine	3.11±0.05	4.69±0.3	5.23±0.23	6.32±0.14
76	2,3,5-Trimethylpyrazine	108.22±4.91	94.55±2.95	106.25±4.18	113.45±3.02
77	2-Ethyl-3,5-dimethylpyrazine	9.5±0.61	8.21±0.28	10.58±0.64	11.89±0.14
78	5-Ethyl-2,3-dimethylpyrazine	3.51±0.41	2.54±0.09	3.28±0.2	3.68±0.04
79	2-Pentylfuran	38.02±1.44	110.79±1.35	133.35±3.76	124.13±7
80	Dimethyl disulfide	25.31±1.44	ND	ND	ND
81	Dimethyl trisulfide	ND	42.18±5.02	60.03±68.84	16.06±2.13
82	Methionol	ND	207.77±9.78	218.74±2.82	213.07±2.13
83	Styrene	8.36±0.38	157.62±20.72	226.75±28.35	216.14±19.38
84	β-Caryophyllene	11.65±0.37	24.2±0.33	16.4±0.64	17.82±1.11

85	Valencene	3.8±0.13	5.79±0.18	3.62±0.11	4.78±0.07

Values are presented as mean ± SD (n = 3). ND, not detected (analyte below detection limit, not equivalent to true zero concentration). ND values were set to zero for PCA, PLS-DA and correlation calculations; 0D, 2D, 14D, and 35D denote fermentation days 0, 2, 14, and 35.

Table S3. Semi-quantitative levels of volatile compounds detected in indica-rice fermented grains (IR) during fermentation.

No.	Aroma compounds	Concentration (μg/g)			
		IR-0D	IR-2D	IR-14D	IR-35D
1	Propyl acetate	ND	3475.76±134.25	ND	ND
2	Isobutyl acetate	ND	182.74±3.67	268.55±0.98	235.15±24.84
3	Ethyl butanoate	ND	299.9±6.66	636.02±12.6	636.71±26.71
4	Ethyl 2-methylbutyrate	ND	141.17±2.77	1121.24±6.43	2077.27±56.37
5	Ethyl 3-methylbutanoate	ND	80.99±6.08	362.68±18.16	531.25±3.89
6	Isoamyl acetate	ND	344.8±13.03	847.06±29.56	860.22±28.56
7	Ethyl valerate	ND	114.24±5.87	328.76±23.17	385.13±12.86
8	Ethyl isocaproate	ND	ND	21.44±0.02	25.84±0.78
9	Ethyl hexanoate	5.54±0.05	1558.25±30.41	3028.74±61.44	3150.8±201.8
10	Ethyl heptanoate	ND	32.66±4.28	177.8±234.5	59.39±6.11
11	Ethyl lactate	ND	ND	1551.34±49.53	2522.34±8.25
12	2-Ethylhexyl acetate	ND	ND	8.61±0.26	8.52±0.37
13	Octyl acetate	31.75±1.16	29.6±0.63	35.52±1.27	39.34±0.83
14	Ethyl nonanoate	2.1±0.08	43.08±0.63	52.86±1.33	57.86±4.02
15	Ethyl DL-Leucate	ND	53.01±0.91	160.00±0.60	198.4±2.56
16	Ethyl 3-(methylthio)propionate	ND	17.98±0.51	70.91±0.5	82.43±3.87
17	γ-Butyrolactone	ND	280.15±1.36	545.26±11.25	496.47±13.54
18	Ethyl decanoate	0.81±0.08	749.31±32.37	748.7±39.13	747.79±34.68
19	Isoamyl octanoate	ND	ND	36.34±2.66	35.92±0.75
20	Ethyl benzoate	41.98±0.5	110.06±5.07	451.33±13.14	679.65±0.92
21	Linalyl butyrate	7.37±1.1	8.46±0.28	4.85±0.01	4.99±0.34
22	Diethyl succinate	1.41±0.11	160.56±14.74	ND	ND
23	Ethyl phenylacetate	ND	51.77±1	291.12±6.2	393.56±3.8
24	Ethyl nicotinate	ND	8.74±0.22	140.2±3.57	421.39±14.23
25	Ethyl laurate	0.15±0.01	12.92±0.48	17.1±0.12	15.79±0.71
26	γ-Nonalactone	ND	1.33±0.06	2.29±0.04	2.72±0.09
27	Ethyl myristate	1.44±0.04	45.73±0.34	79.71±0.66	79.91±2.23
28	Methyl stearate	0.06±0	1.32±0.04	2.36±0.1	2.8±0.01
29	Ethyl stearate	0.17±0.01	4.98±0.09	8.33±0.32	10.33±0.15
30	Ethyl palmitate	8.65±0.08	ND	ND	ND
31	Acetic acid	ND	3451.67±82.61	5699.36±218.18	5828.22±26.51
32	Isobutyric acid	ND	84.54±6.7	114.09±2.81	116.02±3.12
33	Hexanoic acid	ND	17.16±0.08	17.88±0.79	17.05±0.7
34	Nonanoic acid	ND	ND	ND	0.87±0.02
35	Isobutanol	21.45±1.15	ND	ND	45.65±3.69
36	1-Butanol	ND	210.8±4.52	340.96±5.74	334.06±6.7
37	Isoamyl alcohol	353.17±13.23	ND	ND	ND
38	1-Pentanol	55.49±2.08	56.28±2.53	74.56±2.83	75.97±0.41

39	1-Hexanol	756.76±28.12	1603.95±67.65	1978.96±30.19	1938.81±97.83
40	3-Ethoxy-1-propanol	ND	184.05±8.61	259.76±6.73	256.78±6.4
41	3-Octanol	17.25±0.72	17.27±0.31	21.14±0.87	20.08±1.02
42	1-Heptanol	318.67±4.46	245.5±3.81	182.01±3.34	159.92±8.69
43	2-Ethylhexanol	26.52±2.24	25.81±0.55	26.73±1.74	36±0.82
44	2,3-Butanediol	136.64±2.55	2384.08±120.3	2556.73±50.1	2507.08±16.9
45	1-Octanol	27.62±2.09	103.31±2.28	114.15±3.01	102.48±2.01
46	1-Nonanol	37.73±0.35	78.34±1.45	67.18±0.35	57.77±0.59
47	2,6-Nonadienol	ND	9.85±0.8	ND	ND
48	1-Decanol	ND	18.54±1.2	17.6±0.28	15.69±0.09
49	Guaiacol	0.13±0.03	0.33±0.04	0.43±0.01	0.45±0.01
50	Geraniol	ND	ND	1.56±0.04	1.36±0.03
51	Benzyl alcohol	2.06±0.02	0.78±0.01	0.97±0.04	1.1±0.08
52	Butylated hydroxytoluene	0.22±0.02	0.07±0	0.04±0.01	0.03±0
53	2-Phenylethanol	3.58±0.28	ND	ND	ND
54	Phenol	1.62±0.17	1.41±0.02	1.42±0.02	1.3±0.05
55	4-Ethyl-2-methoxyphenol	ND	5.28±0.18	7.68±0.12	7.39±0.53
56	4-Ethylphenol	ND	0.65±0.01	0.85±0.03	0.78±0.01
57	3-Ethylphenol	ND	0.44±0.03	0.62±0.02	0.58±0.01
58	p-Vinylguaiacol	0.74±0.04	20.12±0.89	23.6±0.67	13.74±0.47
59	1-Tetradecanol	ND	1.92±0.03	2.53±0.17	2.75±0.03
60	4-Methoxybenzyl alcohol	ND	0.61±0.03	0.79±0.02	0.86±0.04
61	1-Hexadecanol	ND	0.2±0	0.23±0.03	0.26±0.01
62	α-Amylcinnamyl alcohol	ND	2.87±0.28	4.28±0.15	4.18±0.18
63	Stearyl alcohol	ND	0.35±0.05	0.48±0.03	0.52±0.03
64	Hexanal	59.76±0.1	43.76±0.24	57.53±0.79	103.5±3.37
65	Heptanal	ND	12.87±1.56	38.45±0.69	55.37±0.74
66	(E)-2-Heptenal	242.69±6.75	489.61±9.36	668.52±25.46	1076.55±28.41
67	Nonanal	73.07±2.84	34.51±2.97	38.74±1.99	35.4±3.82
68	(E)-2-Octenal	ND	ND	4917.21±16.06	4810.41±231.94
69	Benzaldehyde	820.71±5.29	548.58±29.83	286.28±13.62	815.1±21.31
70	Phenylacetaldehyde	ND	658.63±17.7	358.31±19.83	521.02±13
71	Pentadecanal	0.84±0.05	1.44±0.05	1.09±0.09	1.13±0.05
72	Hexadecanal	ND	0.73±0.04	0.53±0.02	0.48±0.04
73	Acetoin	223.17±3.87	1735±4.15	432.52±23.41	330.64±6.2
74	1-Octen-3-one	14.37±2.87	17.61±1.06	30.14±1.66	33.63±2.65
75	6-Methyl-5-hepten-2-one	22.81±2.92	52.51±0.94	46.39±1.1	69.09±1.42
76	Pyridine	7.58±0.95	4.84±3.62	7.35±0.3	7.68±0.3
77	2-Acetylpyrrole	0.07±0	0.08±0.01	0.1±0.01	0.11±0
78	2,5-Dimethylpyrazine	13.89±2.18	45.6±59.71	11.67±0.72	15.07±0.35
79	2,6-Dimethylpyrazine	ND	10.32±0.44	13.98±0.07	14.17±0.6
80	2,3-Dimethylpyrazine	34.54±4.03	24.43±0.95	23.37±1.19	23.37±0.56
81	2-Ethyl-5-methylpyrazine	4.43±0.21	3.5±0.23	4.59±0.18	5.92±0.29
82	2,3,5-Trimethylpyrazine	219.39±4.8	233.3±18.4	263.77±9.43	270.62±5.63

83	2-Ethyl-3,5-dimethylpyrazine	22.14±0.71	28.68±0.55	33.7±0.95	35.71±0.29
84	5-Ethyl-2,3-dimethylpyrazine	8.41±0.48	8.88±0.17	10.44±0.29	11.06±0.09
85	2-Pentylfuran	1.88±0.33	24.83±0.84	29.38±2.37	35.88±0.48
86	Dimethyl disulfide	24.88±3.65	ND	ND	ND
87	Dimethyl trisulfide	ND	24.3±1.02	40.99±0.8	44.34±1.38
88	Methionol	ND	214.16±6.26	241.96±4.12	231.07±4.81
89	Styrene	8.14±0.09	123.15±8.1	182.15±4.97	172.05±11.78
90	β-Caryophyllene	27.43±0.45	24.48±1.11	25.28±0.21	19.77±0.52
91	Valencene	4.23±0.21	4.46±0.32	4.44±0.29	4.38±0.6

Values are presented as mean ± SD (n = 3). ND, not detected (analyte below detection limit, not equivalent to true zero concentration). ND values were set to zero for PCA, PLS-DA and correlation calculations; 0D, 2D, 14D, and 35D denote fermentation days 0, 2, 14, and 35.

Table S4. Semi-quantitative levels of volatile compounds detected in japonica-rice fermented grains (JR) during fermentation.

No.	Aroma compounds	Concentration (μg/g)			
		JR-0D	JR-2D	JR-14D	JR-35D
1	Propyl acetate	ND	3185.5±209.46	ND	ND
2	Isobutyl acetate	ND	98.32±18.78	196.33±23.94	162.06±3.06
3	Ethyl butanoate	ND	350.62±9.1	639.03±30.45	567.75±3.5
4	Ethyl 2-methylbutyrate	ND	167.49±4.07	959.35±45.92	1517.69±78.77
5	Ethyl 3-methylbutanoate	ND	44.85±2.13	202.37±11.29	275.12±3.89
6	Isoamyl acetate	ND	250.87±12.74	526.42±8.77	608.51±17.53
7	Ethyl valerate	ND	163.66±1.1	417.34±15.82	371.38±8.85
8	Ethyl isocaproate	ND	ND	25.71±0.71	26.95±2
9	Ethyl hexanoate	3.05±0.11	339.86±444.6	3807.37±131.35	3286.27±111.95
10	Isoamyl butyrate	ND	ND	11.93±0.51	12.27±1.63
11	Ethyl heptanoate	ND	154.25±2.79	205.42±95.57	80.15±31.22
12	Ethyl lactate	ND	ND	2351.6±6.04	3276.61±16.03
13	2-Ethylhexyl acetate	ND	ND	11.12±0.86	8.25±0.63
14	Octyl acetate	32.74±1.47	24.05±0.97	28.41±1.78	35.51±3.06
15	Ethyl nonanoate	1.26±0.11	47.91±0.98	65.49±1.19	62.6±4.27
16	Ethyl DL-Leucate	ND	45.62±1.32	127.8±5.96	164.06±8.34
17	Ethyl 3-(methylthio)propionate	ND	20.18±1.17	71.4±4.21	71.06±3.19
18	γ-Butyrolactone	ND	218.64±14.57	409.19±11.5	383.92±19.62
19	Ethyl decanoate	0.85±0.04	859.21±4.91	831.84±3.38	845.92±5.83
20	Isoamyl octanoate	ND	ND	29.66±1.18	37.07±2.77
21	Ethyl benzoate	56.23±2.04	219.41±17.88	762.87±9.97	1090.27±69.04
22	Linalyl butyrate	7.54±0.08	13.79±0.8	7.44±0.02	8.25±0.19
23	Diethyl succinate	1.4±0.02	187.99±1.85	ND	ND
24	Ethyl phenylacetate	ND	65.22±2.66	315.64±5.23	418.16±18.96
25	Ethyl nicotinate	ND	9.2±0.25	131.11±0.7	359.01±22.69
26	Ethyl laurate	0.16±0.03	14.76±1.33	13.01±1.9	13.25±0.25
27	2-Phenylethyl acetate	ND	0.9±0.06	ND	ND
28	γ-Nonalactone	ND	1.66±0.03	2.87±0.16	3.79±0.08
29	Ethyl myristate	1.45±0.12	50.42±4.32	65.24±3.36	69.99±4.31
30	Ethyl cinnamate	ND	ND	0.29±0.03	0.39±0.02
31	Methyl stearate	0.04±0.01	1.45±0.11	2.07±0.18	2.23±0.1
32	Ethyl stearate	0.12±0.01	5.33±0.39	7.55±0.7	8.28±0.35
33	Ethyl palmitate	8.63±0.12	ND	ND	ND
34	Acetic acid	ND	3561.65±32.95	4838.16±28.5	5609.39±297.49
35	Isobutyric acid	ND	71.48±2.88	88.31±0.62	90.32±2.91
36	Hexanoic acid	ND	18.71±0.66	19.83±0.57	23.73±1.17
37	Isobutanol	26.84±0.45	ND	ND	46.48±0.53
38	1-Butanol	22.22±1.04	189.88±4.77	351.2±1.31	381.67±6.57
39	Isoamyl alcohol	572.2±13.19	ND	ND	ND
40	1-Pentanol	54.41±5.11	74.12±2.81	85.85±4.57	74.13±4.26

41	1-Hexanol	745.63±27.07	1927.43±18.82	2212.07±37.88	2339.52±117.93
42	3-Ethoxy-1-propanol	ND	200.53±5.7	262.83±2.71	257.83±22.12
43	3-Octanol	27.31±0.83	25.25±3.95	36.69±0.97	40.23±0.99
44	1-Heptanol	386.99±12.46	221.88±16.83	221.89±5.2	204.37±4.6
45	2-Ethylhexanol	28.55±1.28	20.97±0.85	27.85±2.58	28.05±0.48
46	2,3-Butanediol	135.04±6.07	2539.2±167.44	2625.94±3.14	3206.34±167.21
47	1-Octanol	30.92±6.13	110.78±6.03	136.4±13.54	150.08±10.83
48	1-Nonanol	42.4±1.58	82.47±3.82	78.32±3.09	77.05±4.14
49	2,6-Nonadienol	ND	11.58±0.65	ND	ND
50	1-Decanol	ND	22.48±0.37	20.00±0.74	19.32±0.43
51	Guaiacol	0.14±0.02	0.46±0.03	0.51±0.02	0.54±0.02
52	Geraniol	ND	1.41±0.04	1.35±0.04	1.45±0.01
53	Benzyl alcohol	1.93±0.02	1.28±0.14	1.3±0.07	1.4±0.05
54	Butylated hydroxytoluene	0.13±0.02	0.07±0	0.05±0	0.04±0
55	2-Phenylethanol	3.75±0.04	ND	ND	ND
56	Phenol	2.13±0.27	1.83±0.04	1.77±0.13	1.77±0.02
57	4-Ethyl-2-methoxyphenol	0.04±0.02	8.5±0.34	10.33±1.16	10.35±0.34
58	4-Ethylphenol	ND	3.63±0.74	4.04±0.47	4.13±0.15
59	3-Ethylphenol	ND	2.37±0.1	2.8±0.02	3.07±0.11
60	p-Vinylguaiacol	3.91±0.01	31.36±0.16	33.3±0.89	27.54±0.51
61	1-Tetradecanol	ND	2.67±0.04	3.22±0.14	3.48±0.08
62	4-Methoxybenzyl alcohol	ND	0.37±0.03	0.84±0.03	0.92±0.07
63	1-Hexadecanol	ND	0.32±0.01	0.34±0.03	0.37±0.03
64	α-Amylcinnamyl alcohol	ND	2.94±0.19	4.33±0.27	4.33±0.17
65	Stearyl alcohol	ND	0.29±0.03	0.45±0.01	0.48±0.05
66	Hexanal	50.33±0.38	74.56±1.74	93.42±5.11	123.53±13.74
67	Heptanal	ND	10.88±0.27	59.59±2.16	44.74±1.06
68	(E)-2-Heptenal	175.4±1.95	545.85±26.67	1273.9±4.25	1007.53±5.86
69	Nonanal	91.14±1.28	61.97±4.51	72.86±7.6	50.56±1.5
70	(E)-2-Octenal	ND	ND	ND	5013.1±64.91
71	Benzaldehyde	484.07±24	667.7±4.03	323.43±7.95	1166.66±4.77
72	(E)-2-Nonenal	37.45±4.86	ND	ND	ND
73	Phenylacetaldehyde	ND	483.34±20.14	185.02±9.46	273.85±17.6
74	Pentadecanal	0.81±0.03	2.57±0.39	0.98±0.06	1.32±0.03
75	Hexadecanal	ND	0.84±0.13	0.48±0.06	0.49±0.03
76	Acetoin	155.62±3.35	1118.95±20.01	224.77±5.79	260.28±13
77	1-Octen-3-one	29.61±6.65	39.94±5.48	73.85±1.46	54.63±2.79
78	6-Methyl-5-hepten-2-one	26.2±1.36	66.05±5.82	76.87±4.08	66.03±0.82
79	Pyridine	26.84±0.3	17.67±0.36	16.02±0.09	17.76±0.93
80	2-Acetylpyrrole	0.09±0.01	0.15±0.01	0.15±0.01	0.18±0.02
81	2-Methylpyrazine	7.04±0.86	ND	ND	ND
82	2,5-Dimethylpyrazine	16.44±0.37	16.03±2.38	16.69±0.93	17.15±0.22
83	2,6-Dimethylpyrazine	ND	13.04±0.07	25.25±0.32	16.53±0.32
84	2,3-Dimethylpyrazine	31.68±2.18	28.45±0.86	25.73±1.19	25.62±1.76

85	2-Ethyl-5-methylpyrazine	4.58±0.31	5.25±0.48	5.96±0.65	7.25±0.39
86	2,3,5-Trimethylpyrazine	243.51±2.13	283.11±5.31	281.17±6.64	292.2±3.58
87	2-Ethyl-3,5-dimethylpyrazine	24.77±0.36	36.58±0.15	38.19±0.68	42.41±2
88	5-Ethyl-2,3-dimethylpyrazine	7.44±0.15	11.02±0.54	12.04±0.52	13.14±0.62
89	2-Pentylfuran	38.12±1.4	32.95±1.27	61.96±0.38	74.5±1.24
90	Dimethyl disulfide	27.95±2.17	ND	ND	ND
91	Dimethyl trisulfide	13.62±1.75	42.66±2.43	49.47±2	44.53±3.08
92	Methionol	ND	217.44±11.04	231.75±11.1	221.98±1.67
93	Styrene	9.49±0.15	277.88±4.85	320.85±4.59	263.76±14.22
94	β-Caryophyllene	24.82±0.24	35.67±2.84	25.33±3.65	25.77±0.45
95	Valencene	4.84±0.75	9.45±0.39	6.33±0.08	6.41±0.21

Values are presented as mean ± SD (n = 3). ND, not detected (analyte below detection limit, not equivalent to true zero concentration). ND values were set to zero for PCA, PLS-DA and correlation calculations; 0D, 2D, 14D, and 35D denote fermentation days 0, 2, 14, and 35.

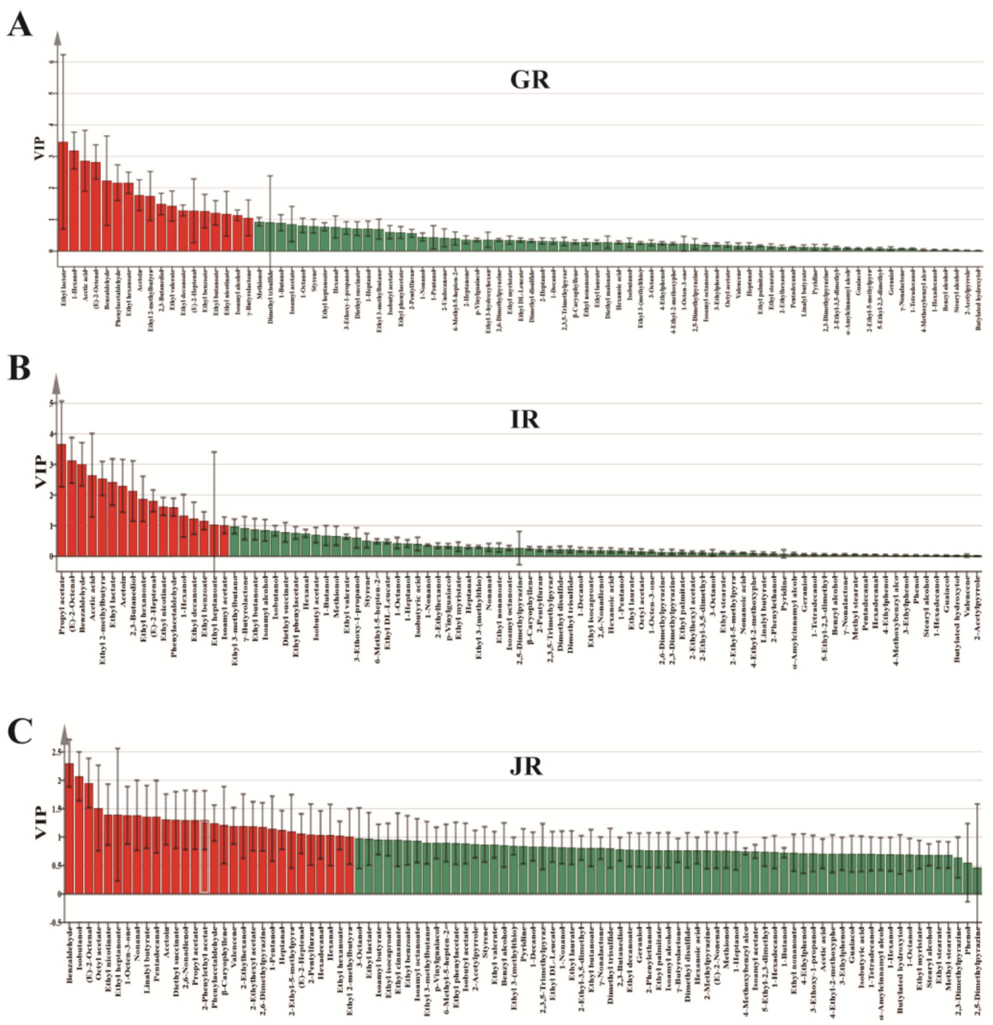

Figure S1. Variable importance in projection (VIP) scores of semi-quantitative levels of volatile compounds contributing to discrimination among fermentation stages in (A) glutinous-rice (GR), (B) indica-rice (IR), and (C) japonica-rice (JR) fermentations, derived from PLS-DA models. The red bar indicates the compound with VIP > 1.

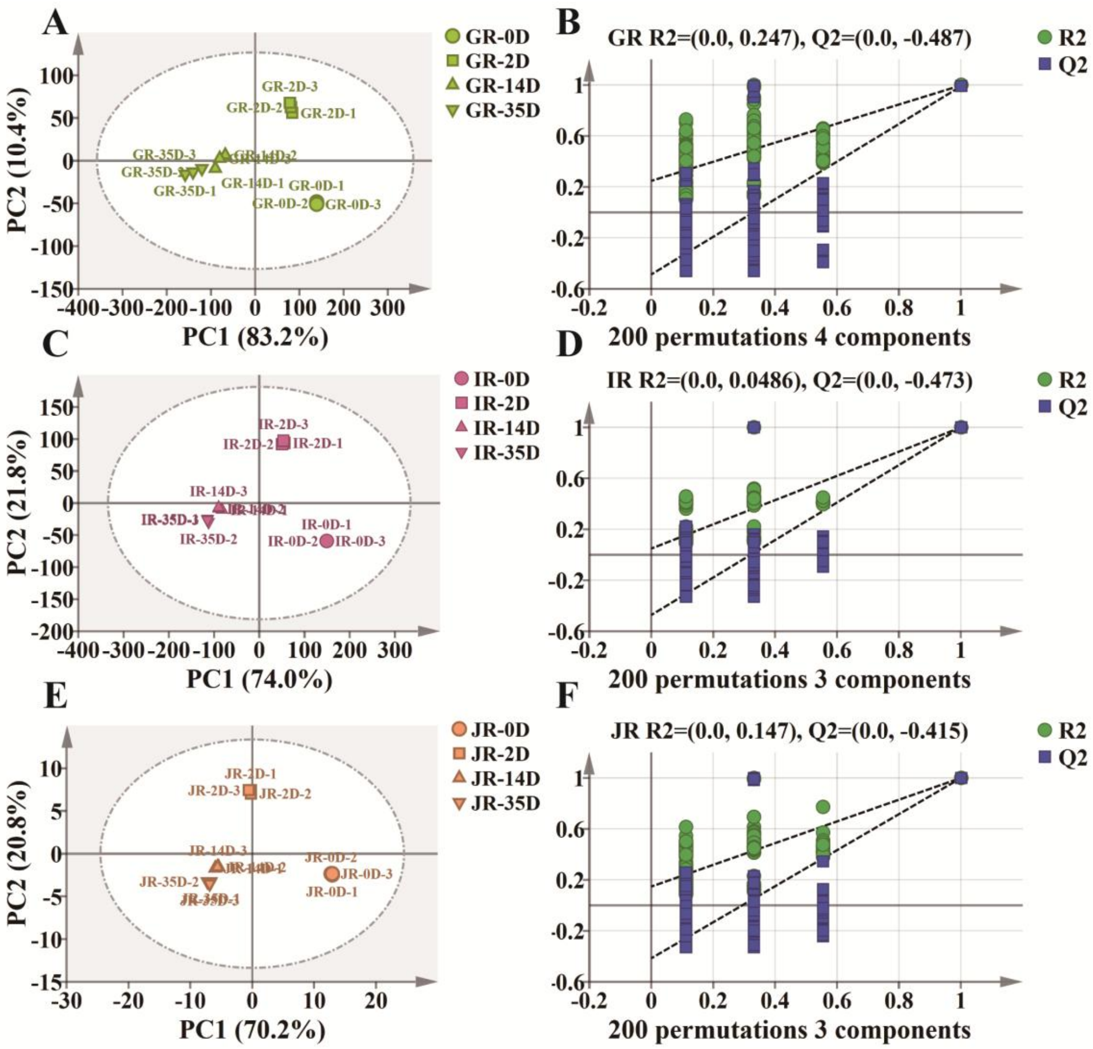

Figure S2. PLS-DA model analysis and permutation test (n = 200) of volatile flavor compounds in fermented grains. Panels A, C, and E show the PLS-DA score plots based on the complete volatile profiles within each rice group; panels B, D, and F show the permutation tests.
